# Molecular subtyping and genomic profiling expand precision medicine in refractory metastatic triple-negative breast cancer: the FUTURE trial

**DOI:** 10.1038/s41422-020-0375-9

**Published:** 2020-07-27

**Authors:** Yi-Zhou Jiang, Yin Liu, Yi Xiao, Xin Hu, Lin Jiang, Wen-Jia Zuo, Ding Ma, Jiahan Ding, Xiaoyu Zhu, Jianjun Zou, Claire Verschraegen, Daniel G. Stover, Virginia Kaklamani, Zhong-Hua Wang, Zhi-Ming Shao

**Affiliations:** 1 Department of Breast Surgery, Fudan University Shanghai Cancer Center; Key Laboratory of Breast Cancer in Shanghai, Shanghai, 200032 China; 2 Department of Oncology, Shanghai Medical College, Fudan University, Shanghai, 200032 China; 3 Jiangsu Hengrui Medicine Co Ltd, Lianyungang, Jiangsu 222002 China; 4 Department of Internal Medicine, The Ohio State University College of Medicine, Columbus, OH 43210 USA; 5 The Ohio State University Comprehensive Cancer Center, Columbus, OH 43210 USA; 6 Division of Hematology/Oncology, University of Texas Health Science Center San Antonio, San Antonio, TX 78284 USA

**Keywords:** Breast cancer, Targeted therapies

## Abstract

Triple-negative breast cancer (TNBC) is a highly heterogeneous disease, and molecular subtyping may result in improved diagnostic precision and targeted therapies. Our previous study classified TNBCs into four subtypes with putative therapeutic targets. Here, we conducted the FUTURE trial (ClinicalTrials.gov identifier: NCT03805399), a phase Ib/II subtyping-based and genomic biomarker-guided umbrella trial, to evaluate the efficacy of these targets. Patients with refractory metastatic TNBC were enrolled and stratified by TNBC subtypes and genomic biomarkers, and assigned to one of these seven arms: (A) pyrotinib with capecitabine, (B) androgen receptor inhibitor with CDK4/6 inhibitor, (C) anti PD-1 with nab-paclitaxel, (D) PARP inhibitor included, (E) and (F) anti-VEGFR included, or (G) mTOR inhibitor with nab-paclitaxel. The primary end point was the objective response rate (ORR). We enrolled 69 refractory metastatic TNBC patients with a median of three previous lines of therapy (range, 1–8). Objective response was achieved in 20 (29.0%, 95% confidence interval (CI): 18.7%–41.2%) of the 69 intention-to-treat (ITT) patients. Our results showed that immunotherapy (arm C), in particular, achieved the highest ORR (52.6%, 95% CI: 28.9%–75.6%) in the ITT population. Arm E demonstrated favorable ORR (26.1%, 95% CI: 10.2%–48.4% in the ITT population) but with more high grade (≥ 3) adverse events. Somatic mutations of *TOP2A* and CD8 immunohistochemical score may have the potential to predict immunotherapy response in the immunomodulatory subtype of TNBC. In conclusion, the phase Ib/II FUTURE trial suggested a new concept for TNBC treatment, demonstrating the clinical benefit of subtyping-based targeted therapy for refractory metastatic TNBC.

## Introduction

Triple-negative breast cancer (TNBC) encompasses a subset of breast cancers that lack expression of the estrogen receptor (ER), progesterone receptor (PR), and human epidermal growth factor receptor 2 (HER2).^1,2^ TNBCs account for 10 to 20% of newly diagnosed breast cancer cases and are associated with higher incidence of visceral metastases, higher risk of early recurrence and worse prognosis.^3,4^

In recent years, the consensus is that TNBC is a highly heterogeneous disease,^5–8^ and this may have implication for TNBC treatment choice. Our previous study presented a multiomic profiling of 465 Chinese patients with TNBCs and provided the largest genomically characterized TNBC dataset to date.^8^ We classified TNBCs into four mRNA subtypes with distinct molecular features: (1) luminal androgen receptor (LAR), (2) immunomodulatory (IM), (3) basal-like immune-suppressed (BLIS), and (4) mesenchymal-like (MES), identified the genomic aberrations that drive each TNBC mRNA subtype, and provided additional insights into TNBC heterogeneity and potential therapeutic options.

Advancement in genomics has fueled the efforts toward “precision oncology”, targeting cancers on the basis of their genetic mutations. Clinical trials focusing on precision oncology are often classified as “umbrella trials”, “platform trials” and “basket trials”. Umbrella trials evaluate multiple targeted therapies for one single disease, such as BATTLE-2 study and Lung-MAP study.^9–13^ However, these studies mainly focused on genomic targets, and did not take molecular subtyping into consideration in the study design. Platform trials set a platform to evaluate multiple targeted therapies for multiple diseases, mainly focusing on refractory solid tumors and rare tumors, such as IMPACT, I-PREDICT and WINTHER platforms.^14–16^ Basket trials refer to designs in which one targeted therapy is evaluated on multiple diseases that have common genetic aberrations. Most precision medicine trials focus on DNA abnormalities, but only a few tumors have tractable genomic alterations. There is an urgent need to explore therapeutic targets beyond the identification of genomic driver aberrations.

**Fig. 1:**
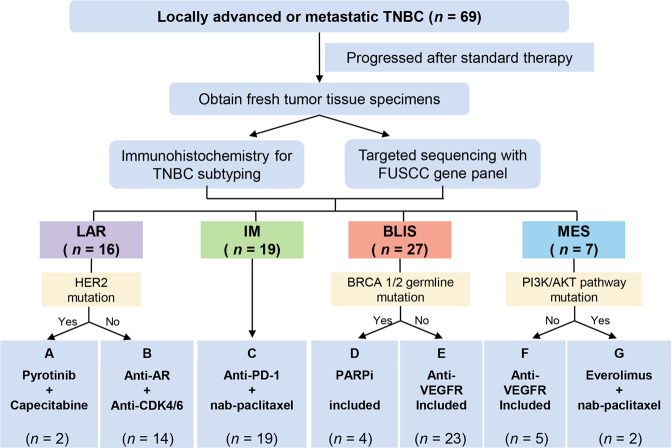
The FUTURE trial schema: integrating TNBC subtyping and genomic targeting. *n*, number of the patients; TNBC, triple-negative breast cancer; FUSCC, Fudan University Shanghai Cancer Center; LAR, luminal androgen receptor; IM, immunomodulatory; BLIS, basal-like immune-suppressed; MES, mesenchymal-like; AR, androgen receptor; PD-1, programmed cell death-1; PARPi, poly ADP-ribose polymerase inhibitor; VEGFR, vascular endothelial growth factor receptor.

Although previous clinical trials have studied several targeted therapies for TNBC, most of these trials were single- or double-arm, and they did not subtype TNBC for a specific target, which may limit the treatment efficacy.^17–21^ Referring to previous umbrella trials, we now present a phase Ib/II Fudan University Shanghai Cancer Center TNBC umbrella (FUTURE) trial, which for the first time combined the TNBC subtyping and genomic sequencing-guided targeted therapy for refractory metastatic TNBC patients (Fig. 1). The FUTURE trial first simplified the TNBC mRNA subtypes by an immunohistochemical (IHC) method using three representative markers. Combining with TNBC IHC subtype-specific genomic features, the FUTURE trial allows most of the patients to enter the corresponding precision treatment arm. We aimed to evaluate the efficacy and safety of multiple precision treatments in heavily pretreated patients with refractory metastatic TNBC who had received a median of three previous lines of therapy. For each enrolled patient, one recurrent or metastatic tumor site was prospectively biopsied for IHC subtyping and targeted sequencing (Supplementary information, Fig. S1 and Table S1). Based on the identified TNBC IHC subtype and genomic biomarkers, patients were then assigned to a certain arm of the study. Here, we report the interim analysis of the FUTURE trial.

## Results

### Patient characteristics

Between October 18th, 2018 and March 25th, 2020, 69 patients were enrolled in the FUTURE trial, out of 87 screened patients (Fig. 1 and Supplementary information, Fig. S2). Table 1 summarizes the clinical characteristics of the 69 enrolled patients. These patients were heavily pretreated (median of three previous antitumor regimens in the metastatic setting (range, 1–8)), most of whom received taxanes (99%), anthracyclines (86%), platinums (88%), vinorelbine (81%), capecitabine (75%), and gemcitabine (72%) before enrollment. Other characteristics were as follows: 81% had two or more metastatic organs, and 67% experienced disease progression within 6 months of their first-line chemotherapy (Table 1), reflecting the heavy tumor burden and resistant disease of the enrolled patients. Detailed patient information is listed in Supplementary information, Table S2.

**Table 1:** Baseline characteristics of the enrolled patients.

Characteristics	Patients (*n* = 69)
Median age at enrollment — year (range)	
	51 (28–74)
ECOG — no. (%)	
0	3 (4%)
1	54 (78%)
2	12 (17%)
Previous lines of treatment — median no. (range)	
	3 (1–8)
Previous use of taxanes or anthracyclines for metastatic or nonmetastatic disease — no. (%)	
Taxanes	68 (99%)
Anthracyclines	59 (86%)
Previous use of chemotherapy drugs for metastatic disease — no. (%)	
Platinum agents	61 (88%)
Gemcitabine	50 (72%)
Capecitabine	52 (75%)
Vinorelbine	56 (81%)
Others	15 (22%)
No. of metastatic organ — no. (%)	
1	13 (19%)
2	26 (38%)
3+	30 (43%)
Metastatic site — no. (%)	
Lymph nodes	43 (62%)
Lung	35 (51%)
Liver	21 (30%)
Bone	30 (43%)
Chest	22 (32%)
Breast	15 (22%)
Others	14 (20%)
Progression-free interval of the first-line therapy (months) — no. (%)	
< 3	24 (35%)
3–6	22 (32%)
> 6	8 (12%)
Unknown	15 (22%)

*ECOG*, Eastern Cooperative Oncology Group.

The median duration of follow-up at the time of cutoff (April 7th, 2020) was 9.2 months (interquartile range (IQR) 5.3–12.2 months). At the data cutoff time, 9 (13.0%) of 69 patients remained on treatment. 40 (66.7%) of the 60 patients discontinued the study due to disease progression. Three (5.0%) patients discontinued the study as a result of serious adverse events. Fourteen (23.3%) patients withdrew from the study. In addition, two (3.3%) patients were lost to follow-up before the first post-baseline tumor assessment, and one (1.7%) patient had a ruptured chest wall lesion as her only site of measurable disease but could not be measured with imaging examination (Supplementary information, Fig. S2).

### Efficacy

**Fig. 2:**
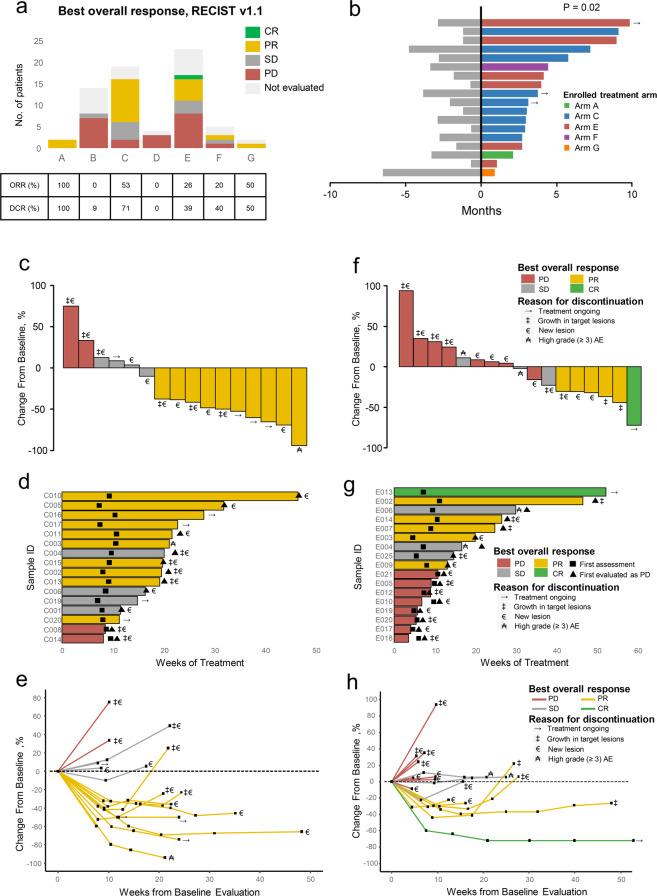
Summary of therapy response. **a** Summary of the category of the best response in each arm of the FUTURE trial. **b** Duration of treatment in the FUTURE and of the last previous therapy in 18 patients in the PP population with available previous treatment duration information. **c**, **f** Best percentage change from baseline in the sum of the longest diameters of target lesions in arm C (**c**) and arm E (**f**). **d**, **g** Time to and durability of treatment in arm C (**d**) and arm E (**g**). **e**, **h** Longitudinal change from baseline in the sum of the longest diameters of target lesions in arm C (**e**) and arm E (**h**). CR, complete response; PR, partial response; SD, stable disease; PD, progressive disease; AE, adverse event.

Patients were enrolled into the following arms based on their TNBC subtypes and genomic features: (A) pyrotinib with capecitabine, (B) androgen receptor inhibitor with CDK4/6 inhibitor, (C) anti PD-1 with nab-paclitaxel, (D) PARP inhibitor included, (E) and (F) anti-VEGFR included, or (G) mTOR inhibitor with nab-paclitaxel. Treatment efficacy of each arm is illustrated in Fig. 2a. Detailed imaging information from representative samples are illustrated in Supplementary information, Fig. S3. Fifty of the 69 enrolled patients underwent at least one post-baseline assessment. In general, objective response (complete response (CR) + partial response (PR)) was achieved in 20 (29.0%, 95% CI: 18.7%–41.2%) of 69 intention-to-treat (ITT) patients and in 20 (40.0%, 95% CI: 26.4%–54.8%) of 50 per-protocol (PP) patients. Disease control (CR + PR + stable disease (SD)) was achieved in 29 (42.0%, 95% CI: 30.2%–54.5%, in ITT population; 58.0%, 95% CI: 43.2%–71.8%, in PP population, respectively) patients. In the 18 patients whose data were available, we compared the duration of treatment provided by the FUTURE trial with that of the patient’s most recent anti-cancer treatment before enrollment. The median duration of treatment provided by FUTURE trial was 3.5 months, whereas that of previous anti-cancer treatment was 2.4 months (*P* = 0.02, Fig. 2b).

Nineteen patients enrolled in arm C were assigned to the treatment of immune checkpoint inhibitor (ICI) plus nab-paclitaxel, with sixteen (84.2%) patients undergoing at least one post-baseline assessment, one (5.3%) patient was in poor physical condition, and was unable to travel to the hospital for assessment, one (5.3%) patient was lost to follow-up, and one (5.3%) patient had a ruptured chest wall lesion that could not be measured with imaging examination (Supplementary information, Fig. S2). Fig. 2c shows the waterfall plot of the sixteen evaluable patients with refractory metastatic TNBC in arm C. Ten (62.5%) of the sixteen patients experienced a PR at first post-baseline evaluation. The objective response rate (ORR) of arm C was 52.6% (95% CI: 28.9%–75.6%) in the ITT population and 62.5% (95% CI: 35.4%–84.8%) in the PP population. Of the ten PR patients, median duration of response was 3.1 months (range 1.0–9.1 months; Fig. 2d, e). At the time of data cutoff (April 7th, 2020), six (60.0%) of the ten PR patients had discontinued the treatment after disease progression. One (10.0%) patient had discontinued the treatment as a result of serious adverse event (rupture of hemangioma of head and face). The remaining three PR patients are still being treated, and have thus far received anti PD-1 with nab-paclitaxel for 2.6–6.5 months (Fig. 2d, e).

Twenty-three patients were enrolled in arm E (anti-VEGFR), among whom seventeen (73.9%) patients underwent at least one post-baseline assessment. The waterfall plot demonstrated that six patients in arm E experienced objective response, with one CR and five PR (Fig. 2f). The ORR of arm E was 26.1% (95% CI: 10.2–48.4%) and 35.3% (95% CI: 14.2–61.7%) in the ITT and PP populations, respectively. Of the six patients with an objective response, the median duration of response was 4.1 months (range 1.1–9.8 months; Fig. 2g, h). Five (83.3%) of six patients discontinued treatments after disease progression, one patient with supraclavicular lymph nodes metastasis after five lines of therapy experienced CR after receiving apatinib for 6.6 months (Fig. 2g, h). Details regarding patients who did not undergo evaluation due to numerous reasons are listed in Supplementary information, Fig. S2.

### Toxicity

Supplementary information, Table S3 summarizes the adverse events (AEs) of each arm. The most common treatment-related AEs of any grade were anemia (*n* = 46, 67%), leukopenia (*n* = 41, 59%), neutropenia (*n* = 30, 43%), thrombocytopenia (*n* = 28, 41%), fatigue (*n* = 26, 38%), hypertension (*n* = 18, 26%), proteinuria (*n* = 18, 26%), and elevated alanine aminotransferase (ALT) (*n* = 17, 25%) (Supplementary information, Table S3). Noticeably, except for hematologic high-grade AEs (≥ grade three), most of the other high-grade AEs occurring in arm E was related to apatinib (500 mg). High-grade AEs, including hypertension (*n* = 5, 22%), proteinuria (*n* = 4, 17%), hand-foot syndrome (HFS) (*n* = 4, 17%), leukopenia (*n* = 2, 9%), and elevated ALT (*n* = 2, 9%) were reported in at least two patients of arm E (Table 2). In particular, two patients whose best response were SD in arm E experienced severe HFS and elevated ALT, which resulted in the discontinuation of treatment before disease progression (Fig. 2g, h). Dose reduction occurred in six (40%) of fifteen patients with apatinib monotherapy. In addition, one PR patient in arm C discontinued the ICI therapy because of the rupture of hemangioma of head and face during the treatment, which was considered by the investigator to be drug-related.

**Table 2:** Treatment-related adverse events in arm E.

Adverse event	No. (%)
Any grade occurring in at least two patients — no. (%)	
Fatigue	13 (57%)
Anemia	12 (52%)
Thrombocytopenia	11 (48%)
Nausea	6 (26%)
Weight loss	6 (26%)
Cough	3 (13%)
Oral mucositis	3 (13%)
Neuropathy	3 (13%)
Vomiting	2 (9%)
Diarrhea	2 (9%)
Abdominal pain	2 (9%)
Grade 3–5 occurring in at least one patient — no. (%)	
Hypertension (Grade 3)	5 (22%)
Proteinuria (Grade 3)	4 (17%)
HFS (Grade 3)^a^	4 (17%)
Leukopenia (Grade 3)	2 (9%)
Elevated ALT (Grade 4)^a^	2 (9%)
Neutropenia (Grade 4)	1 (4%)
Chest distress (Grade 3)	1 (4%)
Decreased appetite (Grade 3)	1 (4%)

*ALT*, alanine aminotransferase; *HFS*, hand-foot syndrome.

^a^Shown are the adverse events that caused discontinuation of drug usage.

### Genomic landscape of refractory TNBC

**Fig. 3:**
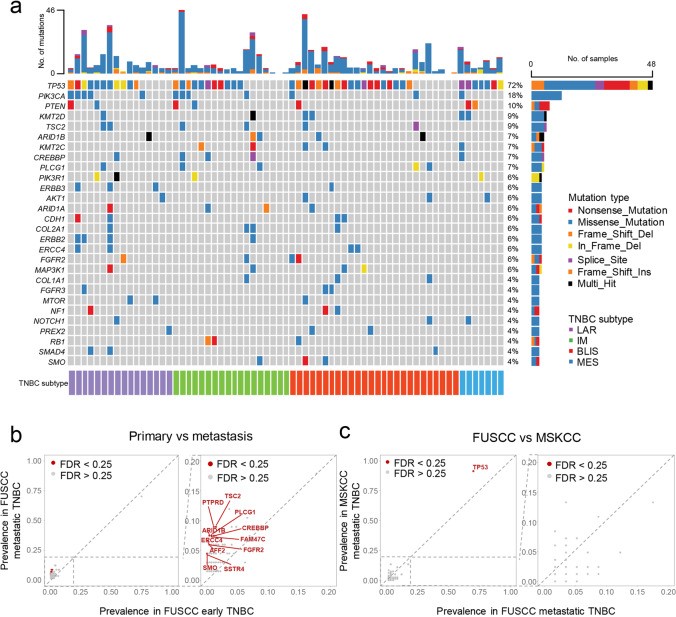
Genomic landscape of refractory TNBC. **a** The genomic landscape of the patients in the FUTURE trial. **b**, **c** Comparison of mutation frequency between primary and metastatic TNBC (**b**) and between FUSCC and MSKCC metastatic TNBC (**c**). MSKCC,  Memorial Sloan-Kettering Cancer Center; FDR, false discovery rate.

In order to describe the genomic landscape of refractory TNBC, we conducted Fudan University Shanghai Cancer Center (FUSCC) next-generation sequencing (NGS) panel of targeted sequencing on the metastatic tumor samples (Supplementary information, Table S4). Most frequently mutated genes in refractory TNBC included *TP53* (72%), *PIK3CA* (18%), *PTEN* (10%), *KMT2D* (9%) and *TSC2* (9%) (Fig. 3a). Metastatic TNBC generally exhibited a similar mutation landscape to their primary counterparts. A higher mutation frequency was observed in some infrequently mutated genes, such as *PTPRD, TSC2, PLCG1, ARID1B, CREBBP* and *FAM47C* (Fig. 3b). We also conducted a race comparison between our FUSCC cohort of Chinese metastatic TNBC and Memorial Sloan-Kettering Cancer Center (MSKCC) cohort of American metastatic TNBC.^22^ A higher mutation frequency of *TP53* was found in the MSKCC cohort (Fig. 3c). No other significant difference was found between the two populations.

### Potential predictors of response in TNBC

**Fig. 4:**
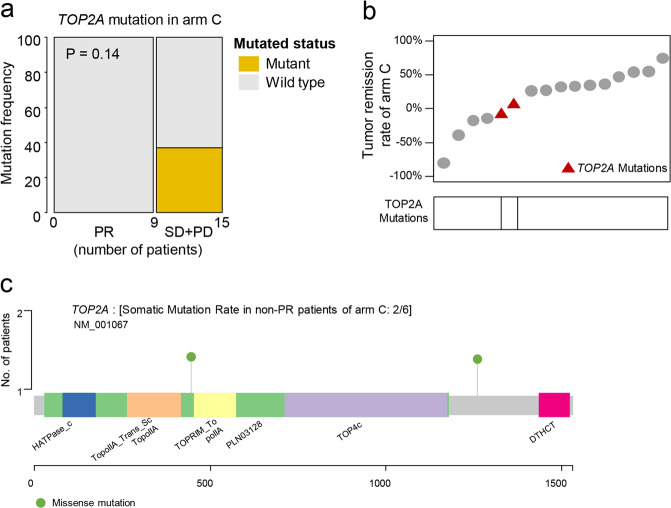
Potential predictors of response for immunotherapy in IM subtype of TNBC. **a** *TOP2A* mutation in PR and non-PR patients of arm C. **b** Relationship between *TOP2A* mutation and tumor remission rate of arm C. **c** The change of amino acid positions related to *TOP2A* mutations in non-PR patients of arm C.

We conducted clinical and genomic analysis to explore the potential predictors of response in refractory TNBC. Firstly, we compared the genomic difference between the patients whose best response was PR versus non-PR in arm C (immunotherapy for IM subtype). *TOP2A* mutation was found in two of the six non-PR patients of arm C, while no mutation was found in PR patients (Fig. 4a, b). One mutation appeared in the domain of DNA topoisomerase 2-like protein (PTZ00108) and the other appeared in the junction part between the two domains of TOP2A protein (Fig. 4c). We also compared the CD8 IHC score (defined as the number of CD8-positive cells divided by the total number of all types of cells on the pathological section of CD8 IHC staining, Supplementary Methods) between progressive disease (PD) and non-PD patients in arm C. Median CD8 score of non-PD patients was 30 while that of PD patients was 22.5 (*P* = 0.25, Supplementary information, Fig. S4c). In addition, PD-L1 IHC score of the immune cells and tumor cells (defined as the number of PD-L1 positive immune cells divided by the total number of immune cells and the number of PD-L1 positive tumor cells divided by the total number of tumor cells on the pathological section of PD-L1 IHC staining, respectively) were evaluated. Some PR patients illustrated higher PD-L1 IHC scores than non-PR patients (Supplementary information, Fig. S4c). Furthermore, we also conducted genomic and clinical analysis in the whole cohort and arm E. A tendency toward higher frequency of drug resistance-related mutations (such as *PTEN*, *RB1* and *NOTCH3)* was observed in PD patients of the whole cohort and arm E (Supplementary information, Fig. S4a, b). There was a tendency of higher FOXC1 IHC score (defined as the number of positive tumor cells divided by the total number of tumor cells on the pathological section of FOXC1 IHC staining, Supplementary Methods) in SD plus PD patients compared with CR plus PR patients in arm E (*P* = 0.26, Supplementary information, Fig. S4c).

## Discussion

This phase Ib/II FUTURE trial confirmed the feasibility of a biopsy-mandated, subtyping-based and genomic biomarker-guided therapy in heavily pretreated refractory metastatic TNBCs. The trial indicated for the first time the potential role of TNBC subtyping and genomic testing in targeted therapy of refractory metastatic TNBCs. Furthermore, the FUTURE trial demonstrated favorable outcomes. The ORR and DCR of the 69 enrolled patients were 29.0% and 42.0%, respectively. Specifically, arms C and E, where the IM and *BRCA1/2* gene wild type-BLIS subtypes were targeted with immunotherapy and anti-VEGFR therapy, respectively, accrued more patients and showed favorable outcomes.

A major focus of the FUTURE trial was to explore the efficacy of anti PD-1 with nab-paclitaxel for TNBCs of the IM subtype. Our previous study revealed that elevated immune cell signaling and tumor infiltrating lymphocytes were hallmarks of the IM subtype. High expression of immune checkpoint genes (such as PD-1, PD-L1, and CTLA-4) suggested potential benefit from ICIs.^8,23^ We applied this hypothesis to arm C of the FUTURE trial, and observed a durable efficacy of ICIs for IM subtype TNBCs, with the ORR of 52.6% (95% CI: 28.9%–75.6%) in the ITT population and 62.5% (95% CI: 35.4%–84.8%) in the PP population. Previous trials of later lines ICI monotherapy in metastatic TNBC demonstrated an ORR around 5%–10%.^24–26^ The ORR increased to around 20% for the first-line monotherapy.^17,27^ Combination of ICI with chemotherapy was also extensively investigated. The KEYNOTE-150 phase Ib/II study evaluated the efficacy of eribulin combined with pembrolizumab.^28^ The ORRs were 29.2% in first-line patients and 22% in second or later lines, respectively. The study initiated by Adams et al. evaluated the clinical efficacy of atezolizumab combined with nab-paclitaxel in metastatic TNBC patients. The ORR was 67% in the first-line, 25% in the second-line, and 29% in the third or later lines.^29,30^ A following first-line phase III study conducted in TNBCs, the IMpassion130 trial, had a response rate of 56% and 58.9% in the ITT population and in the PD-L1-positive subgroup, respectively.^31^ As mentioned above, the target population in our study was much more heavily pretreated, which meant these patients had progressed after using all accessible chemotherapies in the field of breast cancer, including anthracyclines, taxanes, cyclophosphamide, platinums, capecitabine, vinorelbine and gemcitabine. Compared with the trials listed above, the patients in the arm C of the FUTURE trial were enrolled after a median of three lines of therapy, and presented with a heavier disease burden, yet they still achieved a more favorable ORR (Supplementary information, Table S5). Hence, anti PD-1 could be a promising treatment for TNBC with IM subtype.

Furthermore, we revealed that *TOP2A* mutation and CD8 IHC score may be potential predictors of response in the immunotherapy treatment arm. *TOP2A* encodes the topoisomerase (DNA) II Alpha, relieving torsional stress by forming transient double strand breaks of DNA.^32^ Copy number variation and mRNA expression of *TOP2A* were correlated with tumor progression and drug resistance of chemotherapy.^33,34^ However, the mutations of *TOP2A* were less explored. Our study revealed two mutations of *TOP2A* in non-PR patients of immunotherapy. The mutations of *TOP2A* might result in the change of junction peptides between functional domains and might influence the DNA binding of functional domains of TOP2A. Further, we revealed that CD8 IHC staining, which was used in the FUTURE study, might be a practical and feasible methodology for identifying TNBCs with IM subtypes and provide an indication for the administration of ICIs.

Arm E explored the efficacy of VEGFR inhibitor in the treatment of refractory metastatic TNBCs of the BLIS subtype without *BRCA1/2* germline mutation. The ORRs of arm E was 26.1% (95% CI: 10.2%–48.4%) and 35.3% (95% CI: 14.2%–61.7%) in the ITT and PP populations, respectively. A previous clinical trial reported a 10.7% ORR and 25.0% clinical benefit rate for apatinib treatment in heavily pretreated TNBC patients who did not undergo TNBC subtyping,^35^ and various clinical trials regarding bevacizumab have also shown similarly disappointing results.^36,37^ When TNBC patients of the *BRCA1/2* wild-type BLIS subtype were targeted with anti-VEGFR therapy, their responses appeared to be more promising. However, it was noted that the response to anti-VEGFR therapy in these patients was heterogeneous, and was accompanied by significant toxicity from apatinib (500 mg). Due to the frequency of high-grade AEs, the researchers revised the treatment plan of arm E from apatinib 500 mg to apatinib 250 mg (or famitinib 20 mg) plus VP-16 50 mg (Supplementary Methods). The above two issues make us hesitant to perform future trials with apatinib, but encourage further study to uncover other druggable targets for BLIS subtype TNBCs without *BRCA1/2* germline mutation.

Other arms also explored the drug efficacy based on proposed TNBC subtype classification. The LAR subtype was enriched with *HER2* mutations (9%), suggesting a possible benefit with the irreversible tyrosine kinase inhibitor pyrotinib.^8^ At the time of data cut-off, there were two assessable patients in arm A, both of whom achieved a PR after two cycles of therapy. Arms F and G enrolled five and two patients, with the ORRs of 20% and 50% in the ITT population, respectively. However, results from some of the treatment arms were inconsistent with our previous hypothesis. For example, our previous study discovered that LAR subtype TNBCs had less *RB1* losses/deletions, and more frequent *CDKN2A* alterations, which may benefit from CDK4/6 inhibitors.^8^ However, only one of the eight assessable patients in arm B presented with SD at first evaluation, while the remaining seven patients progressed despite being treated with anti-AR combined with anti-CDK4/6 therapy. Interestingly, the genomic analysis showed that all TNBCs in arm B were CDKN2A neutral, which may decrease the efficacy of CDK4/6 inhibitors (Supplementary information, Fig. S5). Genomic landscape of TNBCs in arm B also suggested other potential targets, such as mutations in PI3K-AKT-mTOR pathway. In particular, one TNBC with FGFR1 amplification benefited from anlotinib after PD from anti-AR plus anti-CDK4/6 therapy, and another patient with AR (+) and CD8 (+, 25%) benefited from anti-PD-1 therapy. These results spurs further exploration for therapeutic targets in arm B (Supplementary information, Fig. S6). In addition, arm D tested the hypothesis that the BLIS subtype TNBCs with germline *BRCA1/2* mutation may benefit from PARP inhibitors, but all of the three assessable patients in arm D had progressed at first evaluation (Supplementary information, Fig. S7). We speculate that these inconsistencies may have been due to the small sample size, and three subjects in this arm were platinum-refractory patients.^38,39^ A larger sample size may yield different results, while in the meantime, we need to continue searching for more optimal targets for tumors of this subtype.

We also compared the mutational landscape between the primary and metastatic TNBCs. Some low-frequent mutated genes, such as *PTPRD*, *TSC2*, *PLCG1*, *ARID1B*, *CREBBP* and *FAM47C* were enriched in metastatic samples. *PTPRD* mutation was previously reported in the TCGA cohort,^40^ both in vitro and in vivo experiments confirmed that *PTPRD* acted as a negative regulator of breast cancer metastasis, possibly via downstream IL-6/STAT3 cascade and E2F regulation.^41,42^ *TSC2* was a putative tumor suppressor gene in the PI3K/AKT/mTOR pathway and its mutation was also observed in HR^+^/HER2^−^ metastatic breast cancers in a multi-center cohort.^43^ However, it was also revealed to play a protumorigenic role in breast cancer. High levels of *TSC2* were found to be correlated with increased metastasis in breast cancer patients.^44^ Phosphorylation of *TSC2* was also illustrated to activate the mTORC1 pathway and thus mediated drug resistance toward several targeted therapies of HR^+^ breast cancer.^45–47^ The relationship between *PLCG1* and breast cancer has yet to be studied in depth, with only one research mentioning *PLCG1* expression as a predictor for the *AKT* inhibitor response in vitro.^48^ *ARID1B* was a paralog of frequently mutated tumor suppressor gene *ARID1A*. *ARID1B* overexpression was found to be associated with poor prognosis in TNBC patients, but the mechanism awaits further exploration.^49,50^ *CREBBP* participates in chromosomal remodeling similar to *ARID1A/B* and was identified as a binding protein of CREB. While the relevance between *CREBBP* and breast cancer metastasis has been poorly understood, the CREBBP/β-catenin/FOXM1 axis plays a vital role in TNBC drug resistance via elevating cancer stem cell abundance.^51^ *FAM47C* was a rarely mutated gene and its function remained elusive. In summary, the impact of these infrequently mutated and metastatic-enriched genes remains largely unknown and warrants further exploration.

Owing to the small sample size of this study, some biomarkers, such as *TOP2A* mutation and CD8 IHC score, need further validation. Similarly, further evidence will be required before our efficacy results may be applied upon the general population. However, the majority of targeted therapies featured in our study arms have been reported in other publications, including preliminary results from clinical studies.^17,20,35^ Therefore, the main purpose of this clinical trial was to prove that a combination of TNBC subtyping and genomic sequencing can help screen for patients on whom these targeted treatments would be most effective. The fact that we were able to achieve a favorable efficacy, despite out enrollment of heavily pretreated patients, suggests that our method of screening may greatly benefit the precision treatment of refractory metastatic TNBCs.

Overall, the FUTURE trial has shown that the combination of molecular subtyping and targeted sequencing was a promising treatment strategy for refractory metastatic TNBCs. Current findings of the FUTURE trial would promote further clinical research on precision treatment of TNBCs.

## Materials and methods

### Study population

We recruited patients with refractory metastatic TNBC at FUSCC from October 18th, 2018 to March 25th, 2020. Refractory metastatic TNBC was defined as metastatic TNBC patient who experienced disease progression during or following standard treatment with chemotherapy (including anthracyclines, taxanes, platinums, vinorelbine, capecitabine and gemcitabine). Eligibility criteria included: (1) female patients diagnosed with metastatic breast carcinoma with an ER^−^, PR^−^ and HER2^−^ phenotype. (2) central pathologic examination of tumor specimens performed by the Department of Pathology at FUSCC (ER, PR and HER2 status was independently confirmed by two experienced pathologists based on immunochemical analysis and in situ hybridization). We used < 1% positively stained cells as the cutoff for ER/PR negativity in immunohistochemistry testing according to the American Society of Clinical Oncology/College of American Pathologists Guideline. Eligibility criteria also included having adequate performance status (ECOG grade 0–2) and at least one target lesion suitable for biopsy. Uncontrolled brain metastasis was excluded from the enrollment. Details of the inclusion and exclusion criteria are provided in the study protocol in Supplementary Methods. All patients provided written informed consent. This study was approved by the FUSCC Ethics Committee.

### Study design and oversight

The FUTURE trial is a phase Ib/II, open-label, umbrella trial evaluating the efficacy and safety of multiple targeted treatments based on tumor characteristics of patients with refractory metastatic TNBC. The protocol of this study is available in Supplementary Methods.

As it took nearly one and a half months to receive the sequencing report, the screened patients were suggested to undergo biopsy to provide specimen for TNBC IHC subtyping and molecular tumor-biomarker assessment while being treated with earlier lines of therapy, or at the time of disease progression after the most recent line of standard therapy. Patients who were biopsied and were still undergoing standard chemotherapy could then be enrolled after disease progression. Their pre-obtained specimen-derived testing report would be used for arm assignment.

The samples of baseline tumor biopsy were used to conduct TNBC IHC subtype staining and an FUSCC NGS panel sequencing which detected somatic and germline mutations of 484 breast cancer-specific genes (Supplementary information, Fig. S1 and Table S1). AR, CD8 and FOXC1 were chosen as optimal IHC biomarkers for TNBC subtyping. We considered the differential expression analysis of RNA sequencing data, the correlation between the mRNA and protein expression and the feasibility for IHC in selecting optimal IHC biomarkers. Detailed selection steps are described in Supplementary Methods.

Patients who consented to enroll in the FUTURE trial were assigned to one of these seven treatment arms based upon their subtype and genomic biomarker. The treatment arms included: (A) pyrotinib, a HER2 receptor inhibitor, with capecitabine for the LAR subtype with *ERBB2* somatic mutation, (B) androgen (AR) inhibitor (SHR3680) with CDK4/6 inhibitor (SHR6390) for the LAR subtype without *ERBB2* somatic mutation, (C) anti PD-1 (SH1210) with nab-paclitaxel for the IM subtype, (D) PARP inhibitor (SH3162) included therapy for the BLIS subtype with *BRCA1/2* germline mutation, (E) anti-VEGFR (apatinib or famitinib) included therapy for the BLIS subtype without *BRCA1/2* germline mutation, (F) anti-VEGFR (famitinib) included therapy for the MES subtype without PI3K-AKT pathway mutation, and (G) mTOR inhibitor (everolimus) with nab-paclitaxel for the MES subtype with PI3K-AKT pathway mutation. All the treatment was continued until disease progression, patient withdrawal, or unacceptable toxic effects. Detailed drug usage and dose modifications are provided in Supplementary Methods. This study was registered with ClinicalTrials.gov, number NCT03805399.

### Efficacy evaluation

Computed tomography (CT) and magnetic resonance imaging (MRI) were performed at baseline and at the second cycle after the start of treatment. Subsequent imaging was performed at one or two cycle intervals until disease progression. Assessment of response was performed according to Response Evaluation Criteria in Solid Tumors version 1.1 [RECIST v1.1]. The primary end point of the FUTURE trial was the ORR after two cycles. The secondary end point included DCR, progression-free survival (PFS), overall survival (OS) and safety. ORR was defined as the percentage of patients that experienced CR or PR of the disease. DCR was defined as the percentage of patients that experienced CR, PR or SD. PFS was assessed from the starting date of targeted treatment therapy to the earliest sign of PD or death as a result of any cause. OS was assessed from the starting date of targeted treatment therapy to death as a result of any cause. Duration of response (DOR) was calculated as the date of the first evaluation showing documented PR or CR to the date of the first PD or death, whichever is earlier.

### Safety

Safety evaluations included assessments of AEs and serious adverse events (SAEs), laboratory safety evaluations, vital signs, and physical examination. AEs were assessed in accordance with the National Cancer Institute Common Terminology Criteria for Adverse Events, version 4.0 (CTCAE V4.0). For AEs with various grades, the maximum reported grade was used in Table 2.

### Statistical analysis

The interim analysis was planned to be conducted when 20 subjects were enrolled in at least one arm, and at least one subject was enrolled in each arm, to preliminarily evaluate the efficacy and safety of the drug combination in each arm. With the estimated enrollment speed, around 50% of subjects would have been enrolled by the interim analysis time point. The ITT and PP populations were collected for analysis. The ITT population was defined as all enrolled population. The PP population was a subgroup of patients who were compliant with the protocol and without any major protocol violations. The efficacy of treatment was analyzed in the ITT and PP populations. The patients without at least one post-baseline efficacy evaluation were excluded from the PP population. The ORR and DCR with the 95% CI were calculated with the Clopper–Pearson method. PFS and OS with 95% CI were assessed with the Kaplan–Meier method. Median follow-up time were calculated with the reverse Kaplan–Meier method. Student’s *t*-test, Wilcoxon’s test, and Kruskal–Wallis test were utilized to compare continuous variables and ordered categorical variables. Pearson’s chi-square test or Fisher’s exact test were employed for the comparison of unordered categorical variables. All the tests were two sided, and *P* < 0.05 was regarded statistically significant, unless otherwise stated. R version 3.6.1 (Foundation for Statistical Computing, Vienna, Austria) was used for statistical analysis.

## Supplementary information

Supplementary Methods

Supplementary information, Fig. S1

Supplementary information, Fig. S2

Supplementary information, Fig. S3

Supplementary information, Fig. S4

Supplementary information, Fig. S5

Supplementary information, Fig. S6

Supplementary information, Fig. S7

Supplementary information, Table S1

Supplementary information, Table S2

Supplementary information, Table S3

Supplementary information, Table S4

Supplementary information, Table S5
