# Supplementary material for: Molecular subtyping and genomic profiling expand precision medicine in refractory metastatic triple-negative breast cancer: the FUTURE trial
Source: Cell Res. 2020 Jul 27;31(2):178–86. doi: 10.1038/s41422-020-0375-9 (PMC8027015; doi:10.1038/s41422-020-0375-9)
Supplement: Supplementary file 1 — Supplementary Methods [file 41422_2020_375_MOESM1_ESM.pdf]

1. Supplementary Methods

S1. Biopsy, TNBC subtyping and biomarker testing

S2. Immunohistochemical subtyping of TNBC

S2.1 Selection of markers for IHC-based classification

S2.2 Calculation of the optimal cutoff value of the markers

S2.3 Immunohistochemical staining of samples in the FUTURE trial

S3. Targeted sequencing of the TNBC samples

S3.1 Biospecimen collection, quality control, and processing

S3.2 Targeted sequencing using the FUSCC 484 gene panel

S3.3 Variant calling

S4. Modification of the treatment strategies

S5. Role of the funding source

2. Study protocol

3. Statistical analysis plan

S1. Biopsy, TNBC subtyping and biomarker testing

The procedures of biopsy, TNBC subtyping, and genomic sequencing were as follows: a fresh core needle biopsy of a refractory metastatic tumor lesion was obtained from each patient by an interventional radiologist under computed tomography or ultrasound guidance. Written informed consent was obtained from each patient before the biopsy. Two fresh tumor specimens (approximately 2 cm long each) were collected. One sample was immediately formalin-fixed, then embedded in paraffin, for review by two pathologists at the Department of Pathology of FUSCC for TNBC IHC subtyping. We generally used AR, CD8, and FOXC1 markers to classify TNBC into four subtypes (Supplementary methods and Appendix Figure A1). The other tumor specimen was frozen, stored and allocated for DNA-targeted sequencing. Genomic alterations, such as HER2 somatic mutation, and BRCA1/2 germline mutation, were sequenced based on the FUSCC NGS panel (Supplementary methods and Appendix Table A1). The genomic sequencing was conducted at Chinese National Human Genome Center at Shanghai, which passed the quality control assessment of Chinese government.

S2. Immunohistochemical (IHC) subtyping of TNBC

S2.1 Selection of markers for IHC-based classification

We have previously classified TNBC into four subtypes (BLIS, IM, LAR, MES) in our published paper [1]. Here, we conducted several steps to select optimal TNBC subtype specific markers based on the multi-omics data of our FUSCC TNBC cohort and the TCGA cohort [1]. 1) we performed differential expression analysis on RNA sequencing data using DESeq2 to identify the highly expressed genes ($\log_2$(fold change) > 1.5, adjusted P value < 0.05) in each subtype. 2) using RNA sequencing and mass spectrometry protein expression data from TCGA, we assessed the correlation between the mRNA and protein expression of these genes and retained those that satisfied the following criteria: a correlation coefficient of ≥ 0.5 and a P value of < 0.05. 3) we conducted receiver operating characteristic (ROC) analysis to test the accuracy of using the retained genes to identify the corresponding subtypes and focused on the top 10 genes ordered by the area under the curve (AUC) in each subtype. CD8A and FOXC1 were the top-ranked genes and were therefore selected as markers for the IM and BLIS subtypes respectively. AR was the gene rank fifth but has been demonstrated as a feasible detecting marker and therapeutic target in AR-positive TNBCs [2-4]. Thus, we selected AR as the marker

for the LAR subtype.

S2.2 Calculation of the optimal cutoff value of the markers

We performed IHC staining for both markers on the paraffin-embedded sections. Immunohistochemical staining was performed using an Ventana Benchmark ULTRA automated immunostainer (Ventana Medical Systems, Tucson, Arizona, USA). We used the following primary antibodies: anti-AR (Abcam, ab133273, 1:200 dilution), anti-CD8 (SP57, Ventana, undiluted), anti-FOXC1 (Abcam, ab227977, 1:500 dilution) and anti-DCLK1 (Abcam, ab109029, 1:100 dilution). Since immunohistochemical staining of AR and FOXC1 was mainly found in tumor cells, we measured the protein expression levels of these three markers as the percentage of positive tumor cells (the number of positive tumor cells divided by the total number of tumor cells). By contrast, CD8 staining was primarily found in tumor-infiltrating lymphocytes (TILs), so we measured the protein expression level of CD8 as the percentage of positive cells (the number of positive cells divided by the total number of all types of cells). All stained paraffin-embedded sections were independently evaluated by two experienced pathologists who were blinded to the patients' clinical information. According to the Youden's index of each marker, we employed a cutoff of ≥ 10% positive tumor cells to define AR and FOXC1 positivity, and another cutoff of ≥ 20% positive cells to define CD8 positivity.

S2.3 IHC staining and IHC score definition in the FUTURE trial

We performed IHC staining on paraffin-embedded sections (4 μm thick) of tumor specimens from the patients in the FUTURE trial to evaluate the expression of AR, CD8 and FOXC1. As previously described, IHC staining was performed using an Ventana Benchmark ULTRA automated immunostainer (Ventana Medical Systems, Tucson, Arizona, USA) with the following primary antibodies: anti-AR (Abcam, ab133273, 1:200 dilution), anti-CD8 (SP57, Ventana, undiluted), anti-FOXC1 (Abcam, ab227977, 1:500 dilution) and anti-PD-L1 (SP142, Ventana, undiluted). IHC score of AR and FOXC1 were defined as the number of positive tumor cells divided by the total number of tumor cells on the pathological sections of AR and FOXC1 IHC staining, respectively. CD8 IHC score was defined as the number of positive cells divided by the total number of all types of cells on the pathological sections of CD8 IHC staining. PD-

L1 IHC scores of immune cells and tumor cells were defined as the number of PD-L1 positive immune cells divided by the total number of immune cells and the number of PD-L1 positive tumor cells divided by the total number of tumor cells on the pathological section of PD-L1 IHC staining, respectively.

S3. Targeted sequencing of the TNBC samples

S3.1 Biospecimen collection, quality control, and processing

Tumor and matched blood DNA were isolated from fresh frozen biopsy samples and peripheral lymphocytes using TGuide M24 (Tiangen, Beijing, China). The percentage of tumor cells was confirmed to be adequate in the specimens for sequencing. The purity and quantity of total DNA were estimated by measuring the absorbance at 260 nm (A260) and 280 nm (A280) using a NanoDrop 2000 spectrophotometer (Thermo Scientific, Wilmington, DE, USA). The extracted DNA was considered pure and suitable for future experiments if the A260/A280 ratio was within 1.6-1.9.

S3.2 Targeted sequencing using the FUSCC 484 gene panel

A custom-designed genetic panel, which constituted a hybridization-capture-based assay of 484 genes that are targets of approved and experimental therapies as well as frequently mutated genes in breast cancer, was used in this study. The panel was designed for the detection of mutations and small insertions and deletions. In-house generated RNA baits were utilized to capture all protein-coding exons of target genes. The RNA baits were generated from an oligo pool synthesized by Synbio Technologies (Suzhou, China). The detailed laboratory protocol was described previously [5].

Both tumor and matched blood samples were sequenced. At least 10 ng of each DNA sample obtained after SYBR green quantification was fragmented using a Covaris M220 and then subjected to end-repair, A-tailing and adapter ligation using a KAPA HyperPlus kit (Kapa Biosystems) according to the manufacturer’s recommended protocol. Subsequently, 750 ng of prepared DNA in a volume of 3.4 μl was captured by RNA baits, and the captured library was then sequenced using indexing primers. After quantification with a Multi-Mode Reader (BioTek), the libraries were pooled and sequenced using an Illumina HiSeq X TEN platform (Illumina Inc., San Diego, CA, USA).

S3.3 Variant calling

Variant calling and coverage analysis of each capture region were performed using an in-house developed bioinformatics pipeline based on the general variant calling pipeline. Briefly, high-quality reads were mapped to the hg19 version of the human reference genome (GRCh37) using the BWA aligner with the BWA-MEM algorithm and default parameters. The Genome Analysis Toolkit was used to locally realign the BAM files at intervals with mismatched indels and recalibrate the base quality scores of the reads in the BAM files. Germline variants from the blood BAM file were identified using GATK HaplotypeCaller. Somatic mutations were called from the tissue and blood BAM files using GATK4 Mutect2 with the default parameters. The VCF files were annotated using ANNOVAR. The variants and annotation results were transferred into Excel spreadsheets. To improve the specificity, a panel of normal (PON) sample filters was used to filter out expected germline variations and artifacts [1]. Each alteration identified by the pipeline was manually reviewed to ensure that no false positives were reported. Sequencing quality statistics were obtained using SAMtools and GATK.

S4. Modification of the treatment strategies

Modification of the treatment strategies were conducted in arm B D, E and F. In arm E and F, serious adverse events with the relation to the use of apatinib (500mg), such as hypertension and proteinuria, were noticed during the treatment. Therefore, when 3 patients in arm E reached CR or PR, the researchers changed the treatment strategy of arm E and F into apatinib (250mg) with VP-16 or famitinib with VP-16. In arm B and D, the efficacy was not as well as expected, almost all enrolled patients had disease progression at the first post-baseline evaluation. As a result, the researchers modified the treatment strategy of arm B into the follows: B1) for patients with PIK3CA mutation: everolimus with SHR3680; B2) for patients without PIK3CA mutation: SHR6390 with SHR3680. The treatment of arm D was changed into SHR3162 with famitinib. Details of the new treatment strategies were included in the updated study protocol in the supplementary materials.

S5. Role of the funding source

Most of the drugs used in the FUTURE trial was provided by Jiangsu Hengrui Pharmaceuticals Co for free. No marketed drugs in the FUTURE trial (i.e. SHR3680, SHR6390, SH1210, and SH3162) have entered into phase III clinical trials or have submitted for application. The company had no role in study design, data collection, data analysis, data interpretation, writing of the paper, or the decision to submit for publication. The corresponding author had full access to all the data in the study and had final responsibility for the decision to submit for publication.

Reference

1. Jiang, Y.Z.*, et al.* Genomic and Transcriptomic Landscape of Triple-Negative Breast Cancers: Subtypes and Treatment Strategies. *Cancer Cell* **35**, 428-440 e425 (2019).
2. AR Inhibition Achieves Responses in AR(+) Triple-Negative Breast Cancer. *Cancer discovery* **8**, Of8 (2018).
3. Bonnefoi, H.*, et al.* A phase II trial of abiraterone acetate plus prednisone in patients with triple-negative androgen receptor positive locally advanced or metastatic breast cancer (UCBG 12-1). *Annals of oncology : official journal of the European Society for Medical Oncology* **27**, 812-818 (2016).
4. Traina, T.A.*, et al.* Enzalutamide for the Treatment of Androgen Receptor-Expressing Triple-Negative Breast Cancer. *Journal of clinical oncology : official journal of the American Society of Clinical Oncology* **36**, 884-890 (2018).
5. Yang, L.*, et al.* Pathogenic gene screening in 91 Chinese patients with short stature of unknown etiology with a targeted next-generation sequencing panel. *BMC Med Genet* **19**, 212 (2018).

Protocol

This trial protocol has been provided by the authors to give readers additional information about their work.

This supplement contains the following items:

1. Study protocol (page 3-62)

2. Statistical analysis plan (page 63-74)

Precision Treatment of Refractory Triple Negative Breast Cancer Based on Molecular Subtyping

(FUSCC-TNBC- umbrella)

FUTURE Trial

Study Protocol

Study institute: Fudan University Shanghai Cancer Center

Major study investigator: Zhi-Ming Shao, MD. PhD.

ClinicalTrials.gov Identifier: NCT 03805399

Contents

1 Study Background 12

1.1 Current situation and dilemma of treatment of triple negative breast cancer ...12

1.2 Definition of refractory TNBC in this study 12

1.3 Further classification of TNBC based on multi-omics data 13

2 Study Design 18

2.1 Systemic design 18

2.2 Sample size estimation 18

3 Study Purpose...19

3.1 Primary purpose 19

3.2 Exploratory purpose...19

4 Study endpoints 19

4.1 Primary endpoint..19

4.2 Secondary endpoints..20

5 Therapeutic Regimen...20

5.1 The mechanism of drug and supply of drug 20

5.2 Specific regimen 20

5.3 Test compound (details of unlisted drugs as described in annex).......................22

6 Enrollment and Exclusion Criteria 31

6. 1 Enrollment criteria 31

6.2 Common exclusion criteria...33

6.3 Special exclusion criteria for each arm..33

7 Suspension and Exit Criteria..35

7.1 Suspension criteria..35

7.2 Exit criteria...35

8 Research Process and Specific Projects 35

8.1 Baseline examination 35

8.2 During treatment...37

8.3 At the end of treatment 38

8.4 Follow-up 38

9 Dose Adjustment 38

9.1 General dosage 39

9.2 Provisions for treatment suspension 39

9.3 Dose down regulation 39

9.4 Other regulations 40

10 Concomitant Therapy 40

11 Clinical Evaluation 41

11.1 Main endpoints and observation methods 41

11. 2 Secondary endpoints and observation methods 41

12 Treatment of Common Adverse Events 42

12. 1 Treatment of diarrhea 42

12.2 Treatment of hand-foot skin reaction 42

12.3 Treatment of liver injury 43

12.4 Treatment of hypertension 44

12.5 Treatment of proteinuria 45

12.6 Prevention and treatment of stomatitis/oral mucositis/mouth ulcers 45

12.7 Treatment of non-infectious pneumontis 47

12.8 Management of hyperlipidemia and hyperglycemia 49

12.9 Guidelines for supportive care of immune checkpoint inhibitor SHR-1210 or SHR-1316 50

12.10 Management of infusion related reaction 52

13 Management of Complimentary Trial Compounds 54

13.1 Dispensation of compounds 54

13.2 Storage and Management of Compounds 55

13.3 Disposal of Remaining Compounds 55

14 Adverse Events 55

14.1 Definition 55

14.2 Adverse event reporting period 55

14.3 Serious adverse event (SAE) 55

14.4 Report of severe adverse event 56

14.5 Record and report 56

15 Data Processing 58

15.1 Requirements of data filled by investigators 58

15.2 Data traceability and completion of CRF 58

16 Data Set for Statistical Analysis 58

16.1 Analysis data set 58

16.2 Method of statistics 59

16.3 Method of analysis 59

16.4 Statistic expressions 59

16.5 Analysis software 60

16.6 Interim Analysis 60

17 Quality Control and Quality Assurance 60

18 Ethical Principle 60

19 Trials Progress and Data Retention 60

19.1 Trials progress 61

19.2 Data retention 61

20 References 61

Protocol Abstract

<table>
<tr><td>Study title</td><td>Precision Treatment of Refractory Triple Negative Breast Cancer Based on Molecular Subtyping (FUSCC-TNBC- umbrella) FUTURE Trial</td></tr>
<tr><td>Study design</td><td>Prospective, multi center, open, umbrella phase Ib/II study</td></tr>
<tr><td>Primary endpoint</td><td>Objective Response Rate (ORR)</td></tr>
<tr><td>Secondary endpoints</td><td>1) Disease Control Rate (DCR): CR+PR+SD
2) Progression Free Survival (PFS)
3) Overall Survival (OS)
4) Safety: CTCAE v4.0</td></tr>
<tr><td>Protocol</td><td>Patients with refractory triple-negative breast cancer (TNBC) who had progressed after standard treatments (anthracyclines, taxanes, platinums, vinorelbine, capecitabine, and gemcitabine included) were identified as seven treatment arms and six treatment groups based on the different multi-gene expression profiles and potential molecular characteristics of different pathways.
<table>
<tr><td>Arms</td><td>Conditions</td><td>Regimens</td></tr>
<tr><td>A</td><td>LAR subtype → HER2 gene activation mutation</td><td>Pyrotinib 400mg qd and capecitabine 1000mg/m2 bid (d1-d14)</td></tr>
<tr><td>B</td><td>LAR subtype→without HER2 gene activated mutation
B1→with PIK3CA mutation
B2→without</td><td>B1:
Everolimus 10mg p.o qd continuously.
SHR3680 240mg p.o qd continuously.
4 weeks as a cycle
B2:</td></tr>
</table>
</td></tr>
</table>

		PIK3CA mutation	SHR6390 150 mg p.o qd, 3 weeks on, 1 week off.<br>SHR3680 240mg p.o qd continuously.<br>4 weeks as a cycle
	C	IM subtype→TILs high infiltration	SHR1210 200mg, i.vgtt, d1,every 2 weeks<br>Nab-paclitaxel 100mg/m^2 i.vgtt d1,8,15,<br>4 weeks as a cycle
	D	BLIS subtype→BRCA mut +	SHR3162 150mg p.o bid, continuously.<br>Famitinib 20mg p.o qd continuously.<br>4 weeks as a cycle
	E	BLIS subtype→BRCA mut -	E2:<br>Apatinib 250mg p.o qd continuously.<br>VP-16 50mg p.o qd,2 weeks on, 1 week off.<br>3 weeks as a cycle<br>E3:<br>Famitinib 20mg p.o qd continuously.<br>VP-16 50mg p.o qd,2 weeks on, 1 week off.<br>3 weeks as a cycle
	F	MES subtype	Famitinib 20mg p.o qd continuously.

			VP-16 50mg p.o qd,2 weeks on, 1 week off.<br>3 weeks as a cycle
	G	MES subtype→PI3K activation mutation	Everolimus 10mg p.o qd continuously.<br>Nab-paclitaxel 100mg/m^2 i.vgtt d1,8,15,<br>4 weeks as a cycle
Inclusion criteria	1) Age more than 18 years old.<br>2) Histologically confirmed invasive TNBC (specific definition: the positive definition of ER <1% of the tumor cells was defined as ER negative, PR <1% tumor cell positive was defined as PR-negative, HER2 0-1 + or HER2 was + + but was detected as negative by FISH or CISH without amplification, defined as HER2-negative).<br>3) Locally advanced breast cancer or metastatic breast cancer.<br>4) Refractory triple-negative breast cancer (the existing treatment has failed), specific definition: anthracycline, taxanes, platinum, capecitabine, gemcitabine and vinorelabine, the above commonly used chemotherapeutic drugs failed.<br>5) There is at least one measurable lesion according to RECIST version 1.1 standard (conventional CT scan ≥ 20 mm, spiral CT scan ≥ 10 mm).<br>6) The main organ functions are basically normal and meet the following conditions: HB ≥ 90g/L (no blood transfusion). ANC ≥ 1.5 × 10^9/L ≥ 75 × 10^9/L within 14 days), biochemical examination should meet the following criteria: TBIL ≤ 1.5 ×		

	ULN (upper limit of normal value). ALT and AST ≤ 3 × ULN, ALT and AST ≤ 5×ULN if there is liver metastasis). Serum Cr ≤ 1×ULN, endophytic creatinine clearance rate > 50ml/min (Cockcroft-Gault formula).<br>7) No radiotherapy, endocrine therapy, molecular targeted therapy, and surgery were received within 3 weeks prior to the start of the study, and recovered from the acute toxicity response previously treated. no peripheral neuropathy or I-degree peripheral neurotoxicity.<br>8) ECOG score ≤ 2 and life expectancy ≥ 3 months.<br>9) Female subjects with fertility were required to use a medically approved method of birth control during the study treatment and at least three months after the last use of the research drug.<br>10) The subjects volunteered to join the study and signed the informed consent form with good compliance and follow-up.
Exclusion criteria	1) Radiotherapy (except for palliative reasons), chemotherapy, immunotherapy, bisphosphonate (except for bone metastases) three weeks before treatment.<br>2) Uncontrolled central nervous system metastases (symptomatic or requiring the use of glucocorticoids or mannitol to control symptoms).<br>3) History of clinically important or uncontrolled heart diseases, including congestive heart failure, angina pectoris, myocardial infarction or ventricular arrhythmias in the last six months. ongoing adverse reactions ≥1 due to previous treatment. the exception to this is hair loss or what researchers believe should not be excluded. Such

	cases should be clearly documented in the investigator's notes.<br>4) Pregnant or lactating patients.<br>5) Malignant tumors in the past five years (except for cured skin basal cell carcinoma and cervical in situ cancer).
Sample size	Participants will enter different treatment arms according to their molecular subtype (IHC staining) and FUSCC 484 gene panel testing results. Seven treatment arms and six regimens were initially set up. 20 patients are planned to be treated per treatment arm.
Research process	Treatment until the tumor progression or an intolerable toxicity.
Safety evaluation	All adverse events during the study period should be recorded in CRF tables, and the researchers should judge the degree of the adverse events and their relationship with the tested drugs.
Duration of study	30 months (2018.08-2021.02)
Follow up	12 months after the last case was admitted to the study
Main analysis date	2022.02

Precision Treatment of Refractory Triple Negative Breast Cancer Based on Molecular Subtyping

(FUSCC-TNBC- umbrella)

FUTURE Trial

1 Study Background

1.1 Current situation and dilemma of treatment of triple negative breast cancer

As a special molecular classification of breast cancer, triple negative breast cancer (TNBC) refers to breast cancer, which lacks the expression of estrogen receptor (ER), progesterone receptor (PR), and human epidermal growth factor receptor-2 (HER2), accounting for about 15% of the total. It has special molecular expression characteristics, biological behavior and clinicopathological characteristics, showing relatively young age, the risk of early recurrence, high distant metastasis rate, high probability of visceral metastasis and brain metastasis, rapid progress, limited treatment [1-4]. The concept of triple-negative breast cancer has been put forward for nearly 20 years, although there are some basic and clinical research results, there is no breakthrough. A large number of clinical trials try to study the potential therapeutic targets of triple-negative breast cancer. the overall prognosis of triple-negative breast cancer has not improved. Once recurrence and metastasis occur, it indicates that the overall survival time is only about 1 year, and it is still the worst prognosis type in breast cancer.

The reason for the dilemma of TNBC is the heterogeneity [5-7]. The naming of TNBC is an exclusive diagnosis, but it is in fact a group of heterogeneous diseases. The research direction is to distinguish the subtypes of TNBCs, to figure out potential therapeutic targets of each subgroup, to improve the treatment strategy and prognosis of these patients.

1.2 Definition of refractory TNBC in this study

Because of the lack of traditional effective endocrine and anti-HER2 targeted

therapy for triple-negative breast cancer, chemotherapy is the most important systemic treatment for patients with advanced triple-negative breast cancer. The commonly used chemotherapeutic drugs available in China include anthracyclines, taxanes, platinum, capecitabine, gemcitabine and vinorelabine. The overall efficacy of the above conventional chemotherapeutic drugs in the treatment of advanced triple-negative breast cancer is poor. According to the results of several phase 3 clinical trials, the overall survival time of advanced triple-negative breast cancer is about 1 year [8-10]. In this study, refractory TNBC was defined as the failure of all the above chemotherapeutic drugs.

1.3 Further classification of TNBC based on multi-omics data

Because triple-negative breast cancer has high invasiveness, few treatments and poor prognosis, there have been a series of studies trying to evaluate the efficacy of targeted drugs for triple-negative breast cancer, but they have failed to achieve the desired results. One of the possible reasons for the analysis is that the patients in these studies were generally triple-negative breast cancer, without further accurate classification of triple-negative breast cancer, and lack of more accurate classification optimization treatment. These failed studies suggest that smarter and more precise treatment strategies should be targeted therapies for possible molecular drive events in each subtype.

The difficulties and dilemma in the treatment of TNBC have been mentioned above. Just as mentioned before, TNBC is a group of heterogeneous diseases. Great effort has been made to further distinguish the subtypes of TNBC, according to the characteristics and potential treatment targets of different subtypes, to achieve the individualized treatment, and finally to improve the prognosis of patients is the direction of research. At present, there are three main studies on how to further classify TNBC:

1)The study of Lehmann et al., from the Vanderbilt-Ingram Cancer Center, published in Journal of Clinical Investigation,2011[11], further divided TNBC into six subtypes:basal-like1 (BL1),basal-like2 (BL2), immune-modulatory (IM),

mesenchymal (M), mesenchymal stem cell-like (MSL), and luminal androgen receptor positive(LAR). The data showed that BL1, IM and MSL had better prognosis. The median total survival time was about 20 months. BL-2, M and LAR had poor prognosis, and the median survival was only 6-8 months. Further, GE analysis helps to identify TNBC cell line models representative of these subtypes. Predicted "driver" signaling pathways were pharmacologically targeted in these cell line models as proof of concept that analysis of distinct GE signatures can inform therapy selection. BL1 and BL2 subtypes had higher expression of cell cycle and DNA damage response genes, and representative cell lines preferentially responded to cisplatin. M and MSL subtypes were enriched in GE for epithelial-mesenchymal transition, and growth factor pathways and cell models responded to NVP-BEZ235 (a PI3K/mTOR inhibitor) and dasatinib (an abl/src inhibitor). The LAR subtype includes patients with decreased relapse-free survival and was characterized by androgen receptor (AR) signaling. LAR cell lines were uniquely sensitive to bicalutamide (an AR antagonist). These data may be useful in biomarker selection, drug discovery, and clinical trial design that will enable alignment of TNBC patients to appropriate targeted therapies. The team published a research paper at PloS One in 2016[12], using histopathological quantification and laser-capture microdissection to determine that transcripts in the previously described immunomodulatory (IM) and mesenchymal stem-like (MSL) subtypes, and they found that these transcripts were contributed from infiltrating lymphocytes and tumor-associated stromal cells, respectively. Therefore, they refined TNBC molecular subtypes from six into four tumor-specific subtypes (BL1, BL2, M and LAR) and demonstrate differences in diagnosis age, grade, local and distant disease progression and histopathology. Using five publicly available, neoadjuvant chemotherapy breast cancer gene expression datasets, they retrospectively evaluated chemotherapy response of over 300 TNBC patients from pretreatment biopsies subtyped using either the intrinsic (PAM50) or TNBCtype approaches. Combined analysis of TNBC patients demonstrated that TNBC subtypes significantly differ in response to similar neoadjuvant chemotherapy with 41% of BL1 patients achieving a pathological complete response compared to 18% for BL2 and 29% for LAR with 95% confidence

intervals (CIs; [33, 51], [9, 28], [17, 41], respectively).

2)The study of Bursteinet et al., published in Clin Cancer Res,2015 [13], suggested that TNBC is a heterogeneous disease. In their study, RNA and DNA profiling analyses were conducted on 198 TNBC tumors, and finally classified TNBC tumors into four distinct subtypes: (i) luminal androgen receptor (LAR), (ii) mesenchymal (MES), (iii) basal-like immunosuppressed (BLIS), and (iv) basal-like immune-activated (BLIA). Of these, prognosis is worst for BLIS tumors and best for BLIA tumors for both DFS (log-rank test: P = 0.042 and 0.041, respectively) and DSS (log-rank test: P = 0.039 and 0.029, respectively). DNA copy number analysis produced two major groups (LAR and MES/BLIS/BLIA) and suggested that gene amplification drives gene expression in some cases [FGFR2 (BLIS)]. Putative subtype-specific targets were identified: (i) LAR: androgen receptor and the cell surface mucin MUC1, (ii) MES: growth factor receptors [platelet-derived growth factor (PDGF) receptor A, c-Kit], (iii) BLIS: an immunosuppressing molecule (VTCN1), and (iv) BLIA: Stat signal transduction molecules and cytokines.

3) **Fudan University Shanghai Cancer Center Four Subtyping** Shao ZM et al.'s study [14-15] comprehensively analyzed clinical, genomic, and transcriptomic data of a cohort of 465 primary TNBC. PIK3CA mutations and copy-number gains of chromosome 22q11 were more frequent in our Chinese cohort than in The Cancer Genome Atlas (TCGA). We classified TNBCs into four transcriptome-based subtypes: (i) luminal androgen receptor (LAR), (ii) immunomodulatory (IM), (iii) basal-like immune-suppressed (BLIS), and (iv) mesenchymal-like (MES). Putative therapeutic targets or biomarkers were identified among each subtype. Importantly, the LAR subtype showed more ERBB2 somatic mutations, infrequent mutational signature 3 and frequent CDKN2A loss. The comprehensive profile of TNBCs provided here will serve as a reference to further advance the understanding and precision treatment of TNBC. According to our previous data, the characteristics of each type and the potential therapeutic targets of the four subtypes are described below:

(i) Luminal Androgen Receptor subtype (LAR) The prognosis of LAR type was moderate, accounting for 21.5% of the total. To explore potential therapeutic targets of

four TNBC subtypes, we further investigated the distinct genomic alterations in each subtype. Memo analysis results implied that both LAR and other subtypes were affected by genomic alterations in receptor tyrosine kinase and cell cycle pathway, but their activating pattern differed significantly. LAR subtype was featured by highest PIK3CA (37% in LAR vs. 13% in other subtypes, FDR < 0.1) mutations and, while other subtypes harbored more copy number amplifications in KRAS and PIK3CA. Interestingly, we found 4 patients (6% in LAR vs 0% in other subtypes, FDR < 0.1,) harboring ERBB2 non-silent SNVs, including activating mutation V777L (in two patients), D769Y (in one patient) and one L755S mutation inferring ERBB2 activation but also resistance to trastuzumab and lapatinib. We further investigated the expression data of these samples. ERBB2 pathway scores defined by Gene Set Variation Analysis (GSVA) demonstrated that patients with ERBB2 mutations also showed activation in ERBB2 pathway. We also searched the database of our institute and found that within 58 IHC determined androgen receptor positive TNBCs, among which eight had ERBB2 somatic mutations.

Although both LAR subtype and others showed frequent genomic alteration in cell cycle related genes, LAR subtype was featured by frequent CDKN2A alteration (19% in LAR vs 7% in others) and astonishingly no RB1 loss or mutation. Combined genomic and expression analysis also supported the impact of these two events (figure 4c), which had been linked to the sensitivity of CDK 4/6 inhibitor, on mRNA expression. In general, LAR is characterized by 16% enrichment of HER2 mutation, partial CDKN2A/B deletion and normal RB1, which may be sensitive to anti-HER2 targeted therapy, anti-androgen endocrine therapy or CDK4/6 inhibitor, respectively.。

(ii) Immuno-Modulatory subtype (IM) The prognosis of IM type was the best, accounting for 22.8%. Despite the relative superior outcome of IM subtype, still 10% patients in this group experienced recurrence and/or metastasis within 5 years since surgery, which promoted us to investigate the potential of additional treatment in this subtype. This subtype was named for its elevated immune cell signaling observed in gene expression data. We first check the immunogenicity of IM subtype. HE stained slides proved that these group of patients had higher prevalence of both stromal tumor

infiltrating lymphocytes (TILs) and intra-tumor TILs [16-17]. Although mutation load was not significantly higher in IM subtype, Gene Set Enrichment Analysis (GSEA) between the IM TNBCs and other subtypes demonstrated the activation in antigen processing and presentation related pathways. In addition, Combing CIBERSORT and differential expression profiling, we demonstrated that immune activating cell and immuno- stimulators were enriched in IM subtype.

As both clinical and omics features had proved that immune recognition has been activated in IM subtype, the way in which these tumors achieved immune escape were likely to be the recruitment of immune suppressive cells or the activation of immune checkpoint molecules. With CIBERSORT algorithm, we checked that immune suppressive cell numbers were not elevated in IM subtype, while expression profiling demonstrated that immunoinhibitors (especially IDO1) were significantly overexpressed in this subtype, providing rationale for the use of immune checkpoint blockade。

(iii) Basal-Like Immune Suppressed subtype (BLIS) The prognosis of BLIS type was poor, accounting for 36.3%. The HRD (homologous recombination repair defect) score was used to further classify the BLIS subgroup-HRD high score group and HRD low score group, in order to accurately screen those patients who were sensitive to DNA damage such as platinum drugs. The high HRD score was significantly correlated with the mutation of BRCA gene, who might benefit from Olaparib [18]. And the prognosis of these patients was significantly better than that of the patients with low HRD score. However, the conditions of this study have been based on empirical treatment of platinum and failed patients. According to the status of BRCA mutation, the patients with BRCA mutation will be treated with PARP inhibitor with famitinib. If there was no mutation in BRCA gene, patients will be treated with apatinib/famitinib, an inhibitor of VEGFR. Apatinib related efficacy and safety data can be found in the attached researcher's manual.

(iv) Mesenchymal subtype (MES) The prognosis of MES subtype was poor, accounting for 19.4%. Gene expression profiling revealed that MES subtype displayed characteristics of breast cancer stem cells (CSCs). We further focused on the

JAK/STAT3 signaling pathway, which played a crucial role in the maintenance of breast CSCs. We observed higher expression of JAK1 and the most important driver of JAK/STAT3 activation IL-6 in the MES subtype. Besides, an activated or tyrosine phosphorylated STAT3 (pSTAT3) gene signature score defined by Sonnenblick et al. was also higher in MES subtype than in other subtypes. These results indicated upregulated JAK/STAT3 signaling pathway and the potential of STAT3 inhibitor treatment for this subtype.

Based on the deep mining of multi-group data of TNBC, we designed this study for patients with refractory (existing standard chemotherapy failure) TNBC. Participants will enter different treatment arms according to their molecular subtype (by IHC staining) and FUSCC 500+ gene panel testing results. This kind of exploratory clinical trial is commonly known as an umbrella trial. After multi-gene detection, it is more beneficial to the individualized treatment and accurate treatment of TNBC by further understanding the nature and characteristics of each subgroup.

2 Study Design

2.1 Systemic design

This study is a prospective, multi-center, open label, umbrella Ib/II phase clinical study. It is planned to screen 300 to 400 patients with refractory TNBC who have failed in the available treatments (Fig. S1). Based on the different multi-gene expression profiles and the potential molecular characteristics of different pathways, seven treatment arms and six treatment groups were initially set up to eroll 20 patients per treatment arm. Therefore, a total of 140 patients were enrolled in this study.

This study is an exploratory Ib/II phase clinical trial, the main purpose of which is to screen valuable therapeutic arm, in order to develop phase III clinical studies with larger samples.

2.2 Sample size estimation

In this study, 3 or more than 3 of 20 patients in each arm group reached CR or PR, will be defined to reach the study end point.

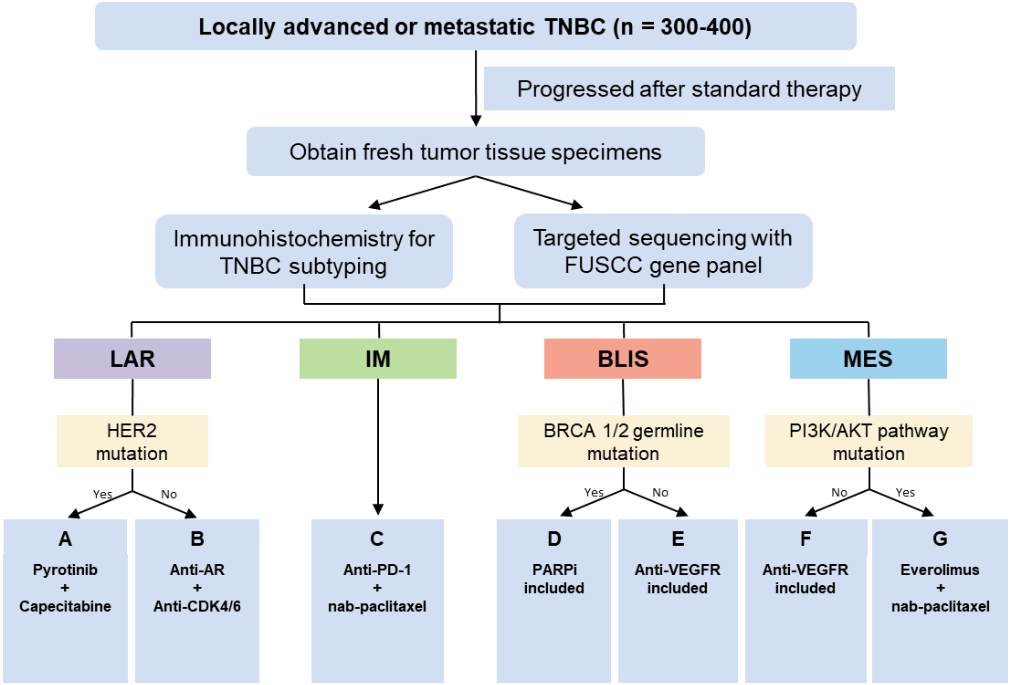

Figure S1. The FUTURE trial schema: integrating TNBC subtyping and genomic targeting

3 Study Purpose

3.1 Primary purpose

To evaluate the efficacy and safety of precision treatment of refractory TNBC based on molecular subtyping.

3.2 Exploratory purpose

To explore the pharmacokinetics of CDK4/6 inhibitor (SHR6390) in combination with AR inhibitor (SHR3680). A total of 18 blood collection points, each blood collection point to take 2 tubes blood (3 ml each), see 8.3 specific description.

4 Study endpoints

4.1 Primary endpoint

Overall Response Rate, (ORR): CR+ PR

4.2 Secondary endpoints

1) Disease Control Rate, (DCR): CR+PR+SD
2) Progression Free Survival, (PFS)
3) Overall Survival, (OS)
4) Safety: CTCAE v4.0

5 Therapeutic Regimen

5.1 The mechanism of drug and supply of drug

Table 1 The drugs used in FUTURE study

name	target	supply
Pyrotinib	Pan-HER inhibitor	Free
Apatinib	VEGFR inhibitor	Free
SHR3162	PRAR inhibitor	Free
SHR6390	CDK4/6 inhibitor	Free
SHR3680	AR inhibitor	Free
SHR1210	Anti PD-1 antibody	Free
Everolimus	mTOR inhibitor	Insurance
Capecitabine	Chemo	Insurance
Albumin-bound nab-paclitaxel	Chemo	Free
Oral etoposide	Chemo	Insurance
Famitinib	VEGFR inhitior	Free

5.2 Specific regimen

The specific treatment options and dosages of the 7 treatment arms in this study are shown in table 2. Participants will receive treatment until disease progression or intolerable toxicity. The evaluation will be based on MRI, CT and physical examination according to novel international standard set by RECIST committee. Evaluation will be done every two cycles (A, E and F arms 6 weeks ±3 days and other arms 8 weeks ±3

days). Since the primary endpoint of this study was total effective, patients who obtained CR or PR required imaging efficacy confirmation after 4 weeks.

In the clinical study process, based on efficacy, safety and existing treatment methods, according to the target and targeted drug availability, new treatment arm may be added during the treatment process, and then it will be submitted to the Ethics Committee for discussion and approval before implementation.

Table 2 Specific medication options for seven treatment arms

Arms	Conditions	Regimens
A	LAR subtype→ HER2 gene activation mutation	Pyrotinib 400mg qd and capecitabine 1000mg/m2 bid (d1-d14)
B	LAR subtype→without HER2 gene activated mutation<br>B1→with PIK3CA mutation<br>B2→without PIK3CA mutation	B1:<br>Everolimus 10mg p.o qd continuously.<br>SHR3680 240mg p.o qd continuously.<br>4 weeks as a cycle<br>B2:<br>SHR6390 150 mg p.o qd, 3 weeks on, 1 week off.<br>SHR3680 240mg p.o qd continuously.<br>4 weeks as a cycle
C	IM subtype→TILs high infiltration	SHR1210 200mg,i.vgtt,d1,every 2 weeks<br>Nab-paclitaxel 100mg/m^2 i.vgtt d1,8,15,<br>4 weeks as a cycle
D	BLIS subtype→BRCA mut +	SHR3162 150mg p.o bid, continuously.<br>Famitinib 20mg p.o qd continuously.<br>4 weeks as a cycle
E	BLIS subtype→BRCA mut	E2:<br>Apatinib 250mg p.o qd continuously.

	-	VP-16 50mg p.o qd,2 weeks on, 1 week off.<br>3 weeks as a cycle<br>E3:<br>Famitinib 20mg p.o qd continuously.<br>VP-16 50mg p.o qd,2 weeks on, 1 week off.<br>3 weeks as a cycle
F	MES subtype	Famitinib 20mg p.o qd continuously.<br>VP-16 50mg p.o qd,2 weeks on, 1 week off.<br>3 weeks as a cycle
G	MES subtype→PI3K activation mutation	Everolimus 10mg p.o qd continuously.<br>Nab-paclitaxel 100mg/m^2 i.vgtt d1,8,15,<br>4 weeks as a cycle

5.3 Test compound (details of unlisted drugs as described in annex)

5.3.1 Pyrotinib

Pyrotinib (SHR1258) is an orally administered dual irreversible tyrosine kinase inhibitor of the epidermal growth factor receptor (HER1) and the human epiderminal growth factor receptor2 (HER2), which is developed by Jiangsu Hengrui Phamaceutical Co LTD. The recommaned dosage of Pyrotinib is 400mg by oral administration everyday.

Pyrotinib has completed phase I and randomized controlled phase II clinical trials in advanced breast cancer in China, and its results have been published in well- known international journals [19-20]. Based on the good tolerance of phase I study, a phase II domestic multicenter clinical study assigned patients with pyrotinib in combination with capecitabine or lapatinib in combination with capecitabine, which has

demonstrated that pyrotinib combined with capecitabine showed superior clinical benefit (ORR and PFS) over lapatinib plus capecitabine. PFS is prolonged more than doubled (18.1months vs. 7.0 months). Due to its excellent efficacy, the drug has entered the CFDA's fast-track designation and will soon be available in China. Two randomized controlled phase III clinical trials of patients with advanced HER2 breast cancer enrollment is almost finished. In addition, there are still many pyrotinib ongoing clinical studies, including the national multicenter randomized controlled double-blind phase III clinical trial of neoadjuvant early HER2 breast cancer leading by FUSCC, and a research on advanced gastric cancer subjects (Research BLTN-Id) and HER2 mutant non-small cell lung cancer Phase II clinical study (HR-BLTN-II-NSCLC study) etc.

Phase II studies have illustrated that pyrotinib combined with capecitabine is well tolerated. Common grade 3-4 toxicity includes hand-foot syndrome 24.6%, diarrhea 15.4%, granulocytopeniaoutu 9.2%, and vomiting 4.6%.

5.3.2 SHR6390

Cyclin-dependent kinase (CDK) 4/6 are important therapeutic targets for breast cancer. Targeted enocrine therapy has shown impressive effectiveness in hormone receptor-positive and HER2-negative (HR + / HER2-) advanced breast cancer recently. First-line combination therapy could achieve 24 months of progression-free survival (for endocrine monotherapy is 14 months), and second-line combination therapy can increase progression-free survival by about 6 months. At present, CDK4 / 6 inhibitors of Pfizer, Novartis and Eli Lilly have all been approved by the FDA for hormone receptor-positive and HER2-negative advanced breast cancer. They have also been recommended by NCCN as the first-class treatment strategy. Palbociclib, a CDK4 / 6 inhibitor developed by Pfizer, was approved by the FDA in 2015 and 2016 respectively, in combination with letrozole or fulvestrant for the treatment of HR + / HER2- advanced breast cancer. Novartis' Ribociclib was also approved by the FDA in combination with aromatase inhibitors to treat HR + / HER2- advanced or metastatic breast cancer in March 2017. Eli Lilly's Abemaciclib was approved for combination with Fulvestrant or monotherapy for HR + / HER2- advanced or metastatic breast cancer that progressed after endocrine therapy in September 2017.

SHR6390 is an oral, highly effective and selective small molecule CDK4 / 6 inhibitor developed by Jiangsu Hengrui Pharmaceutical Co Ltd. In vitro enzymatic tests showed that SHR6390 had a CDK4 / 6 inhibitory activity (IC50 10-12 nM) comparable to Palbociclib, and its selectivity to CDK4 / 6 was much higher than other CDKs (including CDK1 /2/9). In October 2015, SHR6390 successfully obtained the clinical trial approval of China Food and Drug Administration as a new class 1.1 chemical drug. Two Phase I studies intended to explore the tolerability and pharmacokinetics of SHR6390 in advanced solid tumors and melanoma are being conducted in China as of December 2017. Phase I clinical indicated the recommended dose for phase II is 150 mg/d, taken orally once a day for 3 consecutive weeks (D1-21), discontinued at the 4th week (D22-28), and one dose is given every 28 days medicine cycle. Fasting is recommended (fasting should be guaranteed at least 1 hour before and 2 hours after taking the drug during the administration period).

In the above-mentioned Phase I study of advanced solid tumors, among all 20 subjects, most of the drug-related AEs were grades 1-2, grade 3 AEs included neutropenia (7 cases) and leukopenia (5 cases), No grade 4 AE was observed. No withdrew from the study due to AE, and no serious adverse events (SAEs) occurred in this study, which was well tolerated.

5.3.3 SHR3680

SHR3680 is a new oral androgen receptor (AR) inhibitor developed by Jiangsu Hengrui Pharmaceutical Co Ltd. SHR3680 is recommended for oral administration by 240mg once daily.

Preclinical studies have verified that SHR3680 can effectively inhibit AR nuclear translocation, AR transcriptional activity, AR overexpression of prostate cancer cell proliferation and prostate-specific antigen secretion and has no AR partial agonistic effect. SHR3680 is intensively comparable and therefore significantly better than the first generation of AR antagonists bicalutamide.

A phase I/II clinical study designed to explore the tolerability, pharmacokinetics, and efficacy of SHR3680 in patients with advanced castration-resistance prostate cancer (CRPC) has launched in China (study number: SHR- 3680-I / II-CRPC-TOL /

PK / PD). As of April 25, 2017, the dose climb of the Phase I trial has climbed from 40 mg / d to 360 mg / d, accumulating 14 cases, and no dose-limiting toxicity (DLT) has been observed, the Phase II trial is being expanded In the 80, 160 and 240 mg / d dose groups, 45 patients were enrolled. SHR3680 is comfirmed to be safe as shown in preliminary results. Common AEs include hot flashes, male mammary gland development and elevated ALT/AST, all of which are mild to moderate. PSA decreases significantly and bone metastasis can be stabilized. As is demonstrated in the blood samples of the 40, 80, 160, and 240 mg / d dose groups, the pharmacokinetics (PK) showed linear characteristic, with a good absorption and plasma half-life of ~ 4 days. In addition, SHR3680 has also undergone a Phase I clinical study of tolerability and pharmacokinetics in patients with advanced CRPC in Australia. As of April 15, 2017, the study had enrolled 3 patients in the 40 mg / d group and no DLT reported. AE includes abdominal pain, nausea, runny nose, insomnia, gastroesophageal reflux, and diplopia, etc, mostly mild to moderate.

5.3.4 SHR1210

PD-1 is an immune checkpoint protein involved in the negative regulation of antigen-specific T cell function. SHR-1210 (also called INCSHR01210) , a humanized PD-1 antibody, is an original drug independently developed by Jiangsu Hengrui Pharmaceutical Co Ltd. there is a first human trial (FIH) in Australia in addition to 14 ongoing clinical studies in China.

As of November 2017, the most common AE was hemangioma (occurred in> 30% of subjects receiving SHR-1210, especially cutaneous hemangiomas). Other commonly reported AEs (occurring in> 10% of SHR-1210 subjects) include anemia, fatigue, fever, elevated transaminase (alanine aminotransferase [ALT] and / or aspartate aminotransferase [AST]). The most commonly reported (about 1% of subjects) serious adverse events (SAEs) in all studies included hemangiomas, lung infections, pneumonia, upper gastrointestinal (GI) bleeding, liver failure, and malignant progressive tumors. Immune-related SAE pneumonia (occurred in 1.1% of subjects) and interstitial lung disease (occurred in 0.5% of subjects) were regarded as SHR-1210-related adverse events. Other immune-related AEs that occurred in ≥5% of SHR-1210

subjects included elevated AST, elevated ALT, rash, diarrhea, and hypothyroidism.

It is recommended that SHR1210 be a fixed dose of 200 mg, intravenous infusion, once every 2 weeks, every 4 weeks as a cycle.

5.3.5 SHR1316

SHR1316 is an original drug independently developed by Jiangsu Hengrui Pharmaceutical Co Ltd. It is a humanized PD-L1 antibody. As of May 15, 2018, SHR1316 has conducted phase I clinical studies in Australia and China. The Australian study has completed a 20 mg / kg dose group climb. A total of 37 subjects received 4 different dose levels of SHR1316 (1 mg / kg, 3 mg / kg, 10 mg / kg, and 20 mg / kg) and no DLT nor drug-related SAE occurred. Drug-related AEs include diarrhea, hyperthyroidism, headache, nausea, loss of appetite, fatigue, and infusion-related reactions, all are mild or moderate and clinically controllable. A Phase I clinical study (in progress in FUSCC, PI: Professor Hu Xichun) have enrolled 6 subjects (3 in the 3 mg / kg dose group and 3 in the 10 mg / kg dose group) and noDLT or drug-related SAE occurred. The reported drug-related adverse events, including fatigue, abdominal pain, increased bilirubin is mild to moderate, and clinically controllable.

5.3.6 SHR3162

The development of PARP inhibitors could be traced back as early as the 1990s. At first, attempts were made to enhance the efficacy of chemotherapeutic drugs, but the combination of chemotherapeutic drugs and PARP inhibitors proved to be very toxic and research was interrupted. In 2005, two Nature articles made breakthrough progress. The use of PARP inhibitors alone can kill DNA repair defective cancer cells, especially BRCA1 / 2 mutant cancer cells (BRCA participates in DNA repair). Subsequently, AstraZeneca carried out phase I clinical trials of Olaparib (AZD2281), and Sanofi also reported phase II and phase III clinical trials of chemotherapy combined with iniparib for TNBC in 2011. Although it was later confirmed that iniparib is not a true PARP inhibitor, it will produce multiple active fragments in vivo, with a wide range of effects and weak PARP inhibitory effects, but research on PARP inhibitors continues. As of May 2017, a total of 3 PARP inhibitors have been approved by the FDA for the treatment of advanced ovarian cancer: Olaparib of AstraZeneca, Rucaparib of Clovis,

and Niraparib of Tesaro.

SHR3162 (Fluzoparib, fluzoparib) is a class of PARP inhibitors developed by Jiangsu Hengrui Pharmaceutical Co Ltd, which belongs to the 1.1 classification of chemical drugs. As of May 2017, fluzoparil capsules have conducted four phase I clinical studies in China, including a phase I clinical study of tolerability and pharmacokinetics of fluzopalyl in patients with advanced solid tumors (HR-FZPL-I-AST-TOL / PK), an open-dose and dose-climbing phase I study of patinib (FZPL-I-103-GC), an open-dose and dose-climbing phase I study of fluzoparib combined with apatinib (FZPL-I-104 -OC / BC) and a study of food effects and material balance in fluzoparib healthy subjects. The adverse events of fluzoparin include the decrease of hemoglobin, leukocyte, granulocyte and platelet, also fatigue, anorexia, nausea and vomiting.

After a Phase I clinical study, the recommended dose of SHR3162 is 150 mg orally twice daily.

5.3.7 Apatinib

Apatinib (Apatinib, trade name: Aitan) is a small molecule VEGFR tyrosine kinase inhibitor. It blocks the signal transmission after VEGF binding to its receptor by inhibiting the activity of VEGFR tyrosine kinase and inhibit tumor angiogenesis.

Apatinib has undergone Phase I, Phase II of advanced gastric cancer in FUSCC, in which as a leader unit also conducted a national multicenter phase III clinical trial of advanced gastric cancer (marketed clinical). Apatinib was approved for marketing in October 2014 for the treatment of patients with advanced gastric adenocarcinoma or gastric-esophageal junction adenocarcinoma who have progressed or relapsed after receiving at least two types of systemic chemotherapy. Apatinib monotherapy or combined with SHR-1210 for lung cancer, monotherapy or combined with Fluzaparib for gastric cancer, monotherapy or combination with SHR-1210 for liver cancer, monotherapy for colorectal cancer, and Fluzaparib for ovarian cancer and breast cancer, combined with HS10241 in advanced solid tumor clinical research is ongoing.

FUSCC lead two national multi-center Phase II clinical trials for recurrent metastatic breast cancer (TNBC and non-TNBC respectively). The dose for patients

with recurrent metastatic breast cancer is 500 mg orally once daily. The median progression-free survival for apatinib monotherapy for relapsed and metastatic TNBC after multiple courses of treatment was 3.3 months, and the median overall survival was 10.6 months. 3/4-degree hematological toxicity of Apatinib includes thrombocytopenia (13.6%), leukopenia (6.8%), granulocytopenia (3.4%), anemia (1.7%). 3/4-degree non-hematological toxicity includes hand and foot Syndrome (17%), hypertension (11.9%), proteinuria (13.6%) and increased ALT (11.9%).

5.3.8 Everolimus

Everolimus (trade name: Afinitor) derivatives of rapamycin selectively inhibit mammalian rapamycin target protein (mTOR), especially targeting the mTOR-raptor signaling complex. mTOR is a key serine-threonine kinase in the phosphatidylinositol-3-kinase / protein kinase B (PI3K / AKT) signaling cascade and is known to be dysregulated in a variety of human cancers. In January 2013, everolimus was approved for the treatment of advanced RCC in China. In February 2014, everolimus was approved for pNET and TSC-SEGA in China. Everolimus has been approved for oncology indications in more than 105 countries and TSC indications in more than 95 countries.

BOLERO-2, a randomized phase III study shows the efficacy of combined administration of everolimus and exemestane compared with exemestane monotherapy in HR-positive / HER2-negative recurrent metastatic breast cancer after the failure of non-steroidal aromatase inhibitors. There are significant improvements in PFS, response rate and clinical benefit rate. The overall response rate (ORR) of the everolimus + exemestane group versus the exemestane + placebo group (12.6% vs. 1.7%, P <0.0001) and CBR (51.3% vs. 26.4%, P < 0.0001), the median PFS of everolimus + exemestane was 11.0 months, compared with 4.1 months with placebo (HR = 0.38, 95% CI: 0.31-0.48, P <0.0001). The results of the main PFS analysis were confirmed. Based on the results of this randomized phase III study, everolimus and exemestane combination therapy has been approved for marketing in the United States (July 2012), the European Union and many other countries. A subgroup analysis of Asian patients in the BOLERO-2 study showed a median PFS of 8.48 months in the

everolimus plus exemestane group compared to 4.14 months in the placebo plus exemestane group. In Asian patients, the combined treatment reduced the risk of disease progression by 38% compared to exemestane alone. At present, a national multi-centers clinical trial led by FUSCC is ongoing which aimed to prepare for its registration in China. Everolimus dose is 10 mg orally once daily.

The safety profile observed by everolimus in BOLERO-2 is consistent with that previously seen in tumor patients, incidents remain predominantly low-grade (grade 1 or 2). Compared with the control group, an increased risk of non-infectious pneumonia, infection, and stomatitis was observed in the everolimus + exemestane group. The most common adverse events (AE) reported in patients receiving everolimus plus exemestane (≥10% of patients) were stomatitis, rash, fatigue, diarrhea, decreased appetite, weight loss, cough, taste disorders, dyspnea, headache, arthralgia, peripheral edema, anemia, nausea, nasal discharge, vomiting, fever, pneumonia, constipation, back pain, itching, insomnia, weakness, elevated AST / ALT / GGT, hyptuof1erglycemia, xerostomia, alopecia, nasopharyngitis, and urinary tract infections. The most common grade 3-4 AEs suspected of being related to treatment with an incidence of ≥2% are: stomatitis, fatigue, diarrhea, weight loss, dyspnea, anemia, pneumonia, weakness, hyperglycemia and elevated AST / GGT. For advanced HER2-positive breast cancer, there are two randomized phase III double-blind clinical trials (BOLERO-1 and BOLERO-3) that evaluate the efficacy of chemotherapy plus trastuzumab with or without Everolimus as first-line or second-line respectively. The chemotherapeutics selected for these two clinical trials were paclitaxel and vinorelbine. For TNBC, everolimus cannot be combined with endocrine or anti-HER2 drug, so it is feasible to choose everolimus combined with paclitaxel as illustrated on BOLERO-1.

5.3.9 Capecitabine

Capecitabine is a commonly used chemotherapeutic drug for breast cancer and is covered by medical insurance.

5.3.10 Albumin paclitaxel

Albumin paclitaxel is a chemotherapeutic drug approved for breast cancer indications in China and abroad, but its price is relatively high and it has not been

included in the national medical insurance catalog. Patients enrolled in this study require patients who have failed conventional paclitaxel or docetaxel treatment before enrollment. Albumin paclitaxel (domestic or imported) is currently available. In order to reduce the financial burden of patients, the albumin paclitaxel in this study was temporarily Hengrui Company provided it free of charge (it has been marketed in China and has indications for breast cancer). During the course of this study, if the drug enters the national medical insurance catalogue, the drug would no longer be free of charge.

5.3.11 VP-16

VP-16 (also known as Etoposide and Etoposide) is an effective component of lignans isolated from Podophyllin. VP-16 is a cell cycle-specific antitumor drug, acting in late S or G2 phase, and its site of action is topoisomerase II, forming a stable cleavable complex between the three drugs-enzymes-DNA It interferes with DNA topoisomerase II, which makes the damaged DNA irreparable. Topoisomerase II is inserted into DNA to produce the cleavage response required for general cell functions, VP-16 appears to stabilize DNA and topoisomerase II double-strand breaks by stabilizing DNA-cleaving complexes. This product activates certain endonucleases in the body, or acts on DNA through its metabolites, and its non-glycoside homologue 4-desmethylepipodophyllotoxin can inhibit microtubule assembly.

The current indications for VP-16 include first-line treatment of small cell lung cancer (SCLC) and non-small cell lung cancer (NSCLC). In SCLC, the effective rate is 40% -85%, and the complete remission rate is 14% -34%. For NSCLC, the 2013 NCCN guidelines recommend combination of platinum as a first-line chemotherapy regimen. For advanced metastatic breast cancer after multiple courses of treatment, oral VP-16 is listed as one of the optional chemotherapy strategies in the previous NCCN treatment guidelines. Its effective rate is 9% -18%, the median PFS is 3.2 months, but the price is cheap and the oral dosage form is convenient for patients to treat. Adverse reactions include gastrointestinal reactions, allergic reactions, hair loss, etc.

5.3.12 Famitinib

Famitinib (Famitinib malate) is a multi-target tyrosine kinase inhibitor. It has a variety of receptor tyrosine kinases such as VEGFR2, VEGFR3, c-Kit, PDGFRβ, Flt1,

Flt3, and Ret. And c-Src have good inhibitory activity. Preclinical results show that famitinib has obvious curative effects on a variety of human tumors in nude mice transplanted tumors, its antitumor effect is better than similar products Sunitinib in vivo and in vitro.

Currently, famitinib is undergoing phase I and phase II clinical trials in multiple centers in China, involving multiple tumors including advanced intestinal cancer, nasopharyngeal cancer, gastrointestinal stromal tumor, kidney cancer, non-small cell lung cancer, and breast cancer. Phase III clinical trials in advanced colorectal cancer are ongoing, and the drug has not yet been approved for marketing in China. A single arm Phase II clinical result of famitinib in advanced breast cancer that failed multi-line treatment showed that of the 27 patients enrolled in the first stage of Phase II, 4 patients had PR and 2 patients had PR. Confirmed at 4 weeks, ORR was 7.4%, DCR was 22.2%, and median progression-free survival (mPFS) was 1.9 months, suggesting that famitinib has a certain effect on patients with advanced breast cancer. The second phase of a larger sample is underway. In terms of safety, the common hematological adverse reactions of famitinib were mainly decreased in 24 cases (88.9%) of white blood cell count, decreased in 22 cases (81.5%) of neutrophil count, and decreased in 9 cases (33.3%) of platelet count. , Hemoglobin decreased in 2 cases (7.4%). Non-hematological adverse reactions were mainly expressed in 23 cases of proteinuria (48.2%), 21 cases of hand-foot syndrome (77.8%), 14 cases of hypertension (51.8%), 12 cases of fatigue (44.4%), and 6 cases of oral ulcers (22.2 %), 4 cases of stomatitis (14.8%), 12 cases of elevated TSH (44.4%), 8 cases of AKP (29.6%), 7 cases of transaminase (25.9%), 4 cases of TBIL (14.8%)), etc., are common adverse reactions of similar targeted drugs. In this study, most adverse events were mild-to-moderate (degrees I / II), serious adverse events (degrees III / IV) were relatively rare, and no adverse events of degree IV occurred. Famitinib is well toleranted for all subjects.

6 Enrollment and Exclusion Criteria

6. 1 Enrollment criteria

Patients must meet all of the following inclusion criteria to be eligible for this

study

Females ≥18 years of old.

Histologically confirmed invasive TNBC (specific definition: immunohistochemical detection of ER <1% tumor cell positive is defined as ER negative, PR <1% tumor cell positive is defined as PR negative, HER2 0-1 + Or HER2 ++ but negative by FISH or CISH, no amplification, defined as HER2 negative)

Locally advanced breast cancer (cannot be treated with radical local treatment) or recurrent metastatic breast cancer.

Refractory TNBC (existing treatment has failed), specific definition: commonly used chemotherapy drugs available in China include anthracyclines, taxanes, platinum, capecitabine, gemcitabine, and vinorelbine. The above-mentioned commonly used chemotherapeutic drugs has failed. The patient's disease was not effectively controlled or in progress before admission.

At least one measurable lesion according to the RECIST version 1.1 (conventional CT scan ≥20 mm, spiral CT scan ≥10 mm, measurable lesions have not received radiotherapy)

The main organ functions are basically normal and meet the following conditions:

The standard of routine blood test should meet: HB ≥90 g / L (no blood transfusion within 14 days), ANC ≥1.5 × 109 / L, PLT ≥75 × 109 / L.

Biochemical examination must meet the following standards: TBIL ≤1.5 × ULN (upper limit of normal value), ALT and AST ≤3 × ULN, if liver metastases, ALT and AST ≤ 5 × ULN, serum Cr ≤1 × ULN, Creatinine clearance rate> 50 ml / min (Cockcroft-Gault formula)

Have not received radiotherapy, endocrine therapy, molecular targeted therapy, and surgery within 3 weeks before the start of the study, and have recovered from the acute toxicity of previous treatment (if surgery, the wound has completely healed), no peripheral neuropathy or 1 degree peripheral neurotoxicity

ECOG score ≤2, and life expectancy ≥3 months.

Fertile female subjects need to use a medically approved contraceptive during study treatment and at least 3 months after the last use of the study drug.

Subjects volunteered to join the study, signed informed consent, have good compliance, and cooperated with the follow-up.

6.2 Common exclusion criteria

Patients with any of the following were excluded from the study:

Radiotherapy (except for palliative reasons), chemotherapy, and immunotherapy 3 weeks before treatment, except bisphosphonates (can be used for bone metastasis).

Uncontrolled central nervous system metastasis (referring to symptoms or the use of glucocorticoids or mannitol to control symptoms).

A history of clinically important or uncontrolled heart disease, including congestive heart failure, angina pectoris, myocardial infarction or ventricular arrhythmia in the past 6 months.

Adverse reactions with grade ≥1 that are ongoing due to previous treatment. Exceptions to this are hair loss or the investigator's opinion should not be ruled out. Such cases should be clearly documented in the investigator's notes.

Major surgery (except minor outpatient surgery, such as placement of vascular access) within 3 weeks of the first course of trial treatment.

Pregnant or lactating patients.

Malignant tumors in the past five years (except for cured skin basal cell carcinoma and cervical carcinoma in situ).

6.3 Special exclusion criteria for each arm

Special exclusion criteria for arm A

LVEF≥50% (Echocardiography).

Have suffered any of the following heart diseases: (1) Angina pectoris, (2) Arrhythmias requiring medical treatment or clinical significance, (3) Myocardial infarction, (4) Heart failure, (5) Other heart diseases that were considered as not suitable for this study by the investigator.

Special exclusion criteria for arm B

Severe infection within 4 weeks before the first medication (eg, intravenous drip antibiotics, antifungal or antiviral drugs), or fever of unknown origin> 38.5 ° C during screening / before first dose.

Have a history of epilepsy, or a disease that can induce seizures within 12 months before C1D1 (including a history of transient ischemic attack, stroke, brain trauma, and unconsciousness require hospitalization).

Special exclusion criteria for arm C

Subjects who require systemic treatment with corticosteroids (> 10 mg prednisone equivalent daily) or other immunosuppressants within 2 weeks before the first use of study drugs, except for the use of corticosteroids for the prevention of allergies and nausea and vomiting Case. In the absence of active autoimmune disease, inhaled or topical use of steroids and adrenal corticosteroid replacement at doses> 10 mg / day of prednisone are allowed.

Those who have received anti-tumor vaccines or have received live vaccines within 4 weeks before the first administration.

Have a history of active autoimmune diseases, such as interstitial pneumonia, colitis, hepatitis, pituitary inflammation, vasculitis, nephritis, hyperthyroidism, hypothyroidism, including but not limited to These diseases or syndromes). Except for patients with vitiligo or cured childhood asthma / allergies who do not require any intervention in adults, autoimmune-mediated hypothyroidism treated with a stable dose of thyroid replacement hormones, Insulin type I diabetes, asthma patients who need bronchodilators for medical intervention are not included.

Have a history of immunodeficiency, including a positive HIV test, or have other acquired or congenital immunodeficiency diseases, or a history of organ transplantation and allogeneic bone marrow transplantation.

History of interstitial lung disease (except for radiation pneumonia without hormone therapy), history of non-infectious pneumonia.

Subjects had active hepatitis B (HBV DNA ≥ 2000 IU / mL or 104 copies / mL), and hepatitis C (hepatitis C antibody was positive and HCV-RNA was above the lower limit of detection of the analytical method).

Special exclusion criteria for arms E and F

A history of gastrointestinal bleeding or a clear gastrointestinal bleeding tendency within the past 6 months, such as esophageal varices at risk of bleeding, locally active

ulcer lesions, and fecal occult blood ≥ (++) are not eligible for admission, If fecal occult blood (+), a gastroscopy is required.

Abdominal fistula, gastrointestinal perforation, or abdominal abscess occurred within 28 days before participating in this study.

Urine routine shows urinary protein ≥ ++ or confirmed 24-hour urine protein quantification> 1.0 g.

Those who have hypertension and cannot be reduced to normal range with antihypertensive medication (systolic blood pressure> 140 mmHg, diastolic blood pressure> 90 mmHg).

7 Suspension and Exit Criteria

7.1 Suspension criteria

Patients need to stop treatment and continue to be followed up unnder one of the following conditions:

Patients who cannot tolerate after two dose adjustments.

Patients with a delay of dosing for more than 3 weeks.

Disease progress during treastment (PD).

Intolerable adverse reactions occurred during the test.

Pregnancy.

Patients who are not eligible for further treatment due to violation of the research protocol (at the discretion of the investigator).

Any other conditions that investigator consider it necessary to discontinue treatment.

7.2 Exit criteria

Patient should withdraw from this study under one of the following conditions:

Patient withdraws informed consent.

Complicated diseases that seriously affect clinical evaluation.

Any case where the researcher considers it necessary to withdraw from the study.

Receive other systemic treatments or use of drugs forbidden in this study.

8 Research Process and Specific Projects

8.1 Baseline examination

Tissue specimens of recurrent metastatic TNBC (mainly including breast, axillary lymph nodes, lungs, or liver) were re-obtained within 4 weeks before treatment, and the tissues of breast cancer submitted for target seqencing and pathological immunohistochemical detection (ER, PR, HER2, ki67, CK5 / 6, AR, PD-L1, TIL, CD31) in our hospital. Based on the above results, one of the seven treatment arms was selected according to the protocol of this study.

Baseline assessment of CT or MRI of evaluable lesions of the tumor was performed within 3 weeks before treatment.

Baseline records of tumor lesions:

According to the RECIST version 1.1 standard, when there is more than one measurable lesion at baseline assessment, all lesions should be recorded and measured. The total number of lesions does not exceed 5 (each organ does not exceed 2). Of patients selected a maximum of 2 or 4 target lesions as baseline measurement lesions. Target lesions must be selected based on size (longest diameter), can represent all involved organs, and measurements must be reproducible. When the largest lesion cannot be measured repeatedly, a new largest lesion that can be repeatedly measured can be selected again.

Measurable lymph nodes must meet the following criteria: CT measurement of short diameter ≥15 mm. The baseline only needs to detect the short diameter. Usually, the short diameter of the nodule is used to determine whether the nodule has tumor metastasis. The nodule size is generally expressed by two-dimensional data of image detection (CT uses the axial plane, MRI uses the axial plane, Choose one of the sagittal or coronal planes.) The shortest diameter is the minimum value. For example, a 20 mm x 30 mm abdominal nodule with a short diameter of 20 mm can be considered a malignant, measurable nodule. In this example, 20 mm is the measurement of the nodule. Nodules with a diameter of ≥10 mm but less than 15 mm should not be considered as target lesions, nodules less than 10 mm do not belong to the category of pathological nodules and need not be recorded and further observed.

The sum of the diameters of all target lesions (including the longest diameter of non-nodular lesions and the short diameter of nodular lesions) will be reported as the

sum of the baseline diameters. If the lymph node diameter is included, as mentioned above, only the short diameter is counted. The sum of the baseline diameters will be used as a reference value for the baseline level of the disease.

All other lesions, including pathological lymph nodes, can be considered as non-target lesions and do not need to be measured, but should be recorded at baseline assessment, such as "existing", "missing" or in rare cases "clear progress". Extensive target lesions can be recorded with target organs (eg. extensive liver metastases).

Collection of basic data within one week before treatment, including medical history, physical examination, general condition score ECOG score, blood routine, urine routine, liver and kidney function (total bilirubin, ALT, AST, AKP, LDH, total protein, albumin, Urea nitrogen, creatinine, blood glucose), electrocardiogram, hepatitis B (HBV-DNA test if necessary), tumor markers (CEA, CA-153, CA-125). If necessary, check LVEF.

8.2 During treatment

8.2.1 Check the blood routine weekly during treatment and increase the number of tests if necessary.

8.2.2 The blood pressure was measured 3 times a day for 2 weeks before apatinib treatment. If the blood pressure is abnormal, the blood pressure will be monitored daily. If the blood pressure is normal, the blood pressure will be measured twice a week thereafter. Blood and urine routines are performed once a week, liver and kidney function and electrolyte testing, fasting blood glucose monitoring are performed every 2 weeks, AE observation and recording are performed at any time. An electrocardiogram is performed every cycle. If symptoms such as pain in the precardiac area and palpitations occur, the myocardial enzyme spectrum (creatine kinase, lactate dehydrogenase) should be detected immediately, and the electrocardiogram should be checked at any time, and an echocardiogram should be added, One vital sign, PS score, physical examination.

8.2.3 Observe and record patients' clinical symptoms (including appetite, pain) and changes in physical signs. Same as ECOG score.

8.2.4 Record the occurrence and duration of various adverse reactions during

treatment, grade, corresponding treatment, outcome, and judge the relationship with the drug (NCI CTCAE v4.0).

8.2.5 Every two cycles (A arm, E arm, and F arm 6 weeks ± 3 days, other arms 8 weeks ± 3 days) for CT or MRI examination of the relevant lesions, refer to RECIST 1.1 to evaluate the efficacy. Because the primary endpoint of this study was total effectiveness, patients with CR or PR need to be confirmed by imaging at 4 weeks.

8.2.6 The pharmacokinetics evaluations of CDK4 / 6 inhibitor (SHR6390) and AR inhibitor (SHR3680) (Arm B) in the first 6-8 patients were before C1D1 administration, 1 hour after C1D1 administration, 2 hours, 4 hours, 6 hours, 8 hours, 12 hours, before C1D2 administration, before C1D15 administration, before C1D20 administration, before C1D21 administration, 1 hour after C1D21 administration, 2 hours, 4 hours, 6 hours, 8 hours, 12 hours, before the C1D22 administration. Blood Samples were collected a total of 18 times. At each blood collection point, 2 tubes of 3 ml blood were collected for pharmacokinetic parameter evaluation (Hengrui company was responsible for testing).

8.3 At the end of treatment

At the end of treatment: comprehensive tumor assessment, vital signs, physical examination, ECOG score, blood routine, urine routine, electrocardiogram, liver and kidney function, tumor markers (CEA, CA-153, CA-125), and quality of life score.

8.4 Follow-up

The patient entered the post-treatment follow-up period after the last use of the study drug. For patients who were excluded due to non-disease progression, all subjects were followed up from 21 to 35 days after the last dose. Thereafter, they were followed up every 8 weeks, and imaging tests were performed to observe whether the tumor progressed.

During the follow-up period, the following parameters should be recorded: the time of disease progression or death in patients who did not develop disease when taking the study drug, other tumor treatments, SAE related to the study drug, follow-up survival status every 3 months (phone follow-up available).

9 Dose Adjustment

9.1 General dosage

When hematological toxicity reaches III or above or non-hematological toxicity reaches II or above, the investigator decides whether to suspend or reduce the dose. In non-hematological toxicity, nausea, vomiting and fever with a certain cause can be controlled (Such as infections, tumors, etc.), active symptomatic treatment and treatment can be carried out without dose suspension and dose reduction.

9.2 Provisions for treatment suspension

In the treatment, the drug administration should be suspended because the drug toxicity has not recovered. The pause time should not exceed 2 times per cycle to ensure the strength of the drug received by the subjects in the trial.

9.3 Dose down regulation

The drug is poorly tolerated and dose reductions can be performed on the first day of subsequent cycles. Subjects could adjust the dose twice. Once a dose level was lowered, the dose was not allowed to be increased for any reason, but the dose suspension was still allowed. See Table 3 for dose adjustments for each treatment arm.

Table 3 Dose adjustments for each treatment arm

Arm	Drug	Recommended initial dose	First dose reduction	Second dose reduction
A	Pirlotinib	400 mg	320 mg	240 mg
	Capecitabine	1000 mg/m2	750 mg/m2	500 mg/m2
B	SHR6390	150 mg	125 mg	100 mg
	SHR3680	240 mg	160 mg	80 mg
C	SHR1210	200 mg	NA	NA
C	SHR1316	TBD	NA	NA
D	SHR3162	150 mg	100 mg	50 mg

E	Apatinib	Start from 250mg, tolerable dose can be increased to 375mg	250mg	125mg
E、F	Famitinib	20mg	15mg	10mg
E、F	Oral Vp-16	50mg d1-14	50mg d1-12	50mg d1-10
G	Everolimus	10 mg	7.5mg	5mg

9.4 Other regulations

To ensure the consistency of dose adjustment throughout the study, a dose pause was first performed during each dose cycle. After taking the dose suspension measure, if the subject is still poorly tolerated, the dose can be adjusted down on the first day of the next dosing cycle. Suspension of treatment).

10 Concomitant Therapy

In patients who do not have a high risk of infection or a risk of bleeding, it is recommended that colony cell stimulating factor or interleukin-11 or thrombopoietin be administered only in the presence of bone marrow suppression of grade III or higher. Researchers have the right to decide whether they need to deal with it accordingly.

Patients can receive the best supportive care. Patients can receive bisphosphonates for bone metastases during treatment. Complicated clinical diseases and various types of AE should be actively treated, such as rash, diarrhea, hypertension, hand and foot syndrome, liver and kidney function impairment, stomatitis, non-infectious pneumonia, and infusion reactions, etc., according to the judgment of the clinician Give symptomatic treatment. All drugs used in combination should be recorded in the case report form (CRF) in strict accordance with GCP regulations.

This plan prohibits the use of SFDA-approved modern Chinese medicine preparations and immunomodulators (such as thymosin, interferon, interleukin-2, and lentinan) for the treatment of breast cancer.

11 Clinical Evaluation

11.1 Main endpoints and observation methods

Overall Response Rate (ORR):

Defined as the proportion of patients whose tumors shrink to a certain amount and remain for a certain period of time, including cases of CR and PR. The solid tumor remission assessment standard (RECIST version 1.1 standard) was used to evaluate the objective tumor remission. Subjects must be accompanied by measurable tumor lesions at baseline. The efficacy evaluation criteria are divided into complete response (CR), partial response (PR), stable (SD), and progress (PD) according to the RECIST version 1.1 standard (see attachment for details 2).

In this study, 20 patients are prepared for each arm. If 3 patients or more achieved CR or PR, this treatment arm would be considered valuable and reached the main endpoint of this study. It is then worth recommending to design a larger randomized phase III clinical study in the following study project.

11. 2 Secondary endpoints and observation methods

Disease Control Rate (DCR):

Refers to the percentage of patients who can be evaluated for complete response, partial response, and stable disease for more than 4 weeks.

Progression Free Survival (PFS):

Refers to the time between the patient's enrollment and any recorded tumor progression or death from any cause, the analysis of this indicator includes the results of tumor evaluation during study treatment and follow-up. If the patient has several indicators that can be judged as PD, the first indicator to appear when performing PFS analysis, recurrence, new lesions or death are considered to have reached the study endpoint, the patient uses other systemic or anti-target lesions Cancer treatment is also considered PD. For patients who did not have PD or died at the end of the study, the time at which the patient did not show PD for the last time was used as the censored data.

Overal Survival (OS):

Refers to the time from enrollment to death for any reason.

12 Treatment of Common Adverse Events

12. 1 Treatment of diarrhea

Diarrhea: Investigators should inform subjects the possibility of diarrhea and its management before starting treatment. Follow-up and observation (≤14 days) is considered first after onset of diarrhea. Oral montmorillonite powder TID is advised when subjects experiencing diarrhea. Those who experiencing severe diarrhea may be treated with electrolytes solution. Study Medication is withheld until diarrhea improves to Grade≤1 or described as Table 4.

Table 4 Management for diarrhea and guidelines for dose modification for pyrotinib

<table>
<tr><td>CTCAEv4.0</td><td>Management
(After follow-up and symptomatic treatment)</td><td>Dose modification</td></tr>
<tr><td>Grade 4</td><td>Discontinuation</td><td>-</td></tr>
<tr><td>Grade 3</td><td rowspan="2">Study medication withheld until toxicity improves to Grade 0 or Grade 1 with no complication</td><td rowspan="2">Initial Dose:400 mg
First reduction: 320 mg
Final reduction: 240mg</td></tr>
<tr><td>Grade1-2 with complications (including Grade ≥2 nausea or vomiting, fever, hemorrhage or dehydration)</td></tr>
</table>

12.2 Treatment of hand-foot skin reaction

Hand-foot skin reaction (HFSR) is a kind of dermatological adverse event, which may occur as a side effect of certain chemotherapy or targeted therapy. Symptoms of HFSR include numbness, tingling, burning, or itching sensation, swelling, redness, tenderness and rash.

Grade:

Grade 1: Numbness, dysesthesia/paresthesia, tingling, painless swelling or erythema of the hands and / or feet and / or discomfort, which does not disrupt normal

activities.

Grade 2: Painful erythema and swelling of the hands and / or feet and / or discomfort affecting the patient's activities.

Grade 3: Moist desquamation, ulceration, blistering or severe pain of the hands and / or feet and / or severe discomfort that causes the patient to be unable to work or perform activities of daily living.

Management:

Supportive care is considered, including maintaining skin cleanness, avoiding pressure and rubbing, using topical steroid and urea cream, using topical antibiotics when necessary.

Subjects will be withdrawn from this study if they experience HFSR Grade≥2 for 3 times and exacerbate.

12.3 Treatment of liver injury

Elevation of alanine aminotransferase (ALT), aspartate aminotransferase (AST) and total bilirubin (TBIL) may indicate possible drug-induced liver injury (DILI), whose diagnostic threshold is associated with subject's baseline AST/ALT and TBIL level. Medical review is needed to ascertain abnormalities of liver function test (LFT) is not caused by cholestasis, which can be defined as ALP>2×upper limit of normal (ULN) with R<2 in subjects without bone metastasis and hepatic elevation of Alkaline Phosphatase (ALP) in subjects with bone metastasis. R is calculated as ALT/ALP, using multiples of ULN, and it indicates the cause of elevation of ALT and / or ALP, which includes cholestasis, hepatic cell injury and both.

Study medication of subjects with liver injury and without cholestasis should be withheld immediately and check LFT again in 48 hours since abnormalities were found. Complete evaluation should include laboratory tests, medical history, physical examination, possibility of liver metastasis, new hepatic lesions, obstruction or compression.

Hepatic toxicity should include following tests: albumin, ALT, AST, TBIL, Direct Bilirubin (DBIL), Indirect Bilirubin, ALP, creatine phosphokinase (CPK), prothrombin

time (PT) or International Normalized Ratio (INR), γ-Glutamyl transferase (γ-GT). Subjects with Gilbert syndrome must monitor TBIL and DBIL and monitoring closely when DBIL abnormality occurs.

If discontinuation is required because of elevated AST, ALT and / or bilirubin, close observation is recommended, including:

i. Review liver enzymes and serum bilirubin 2 to 3 times a week. If abnormal results are stabilized or recover to normal range, reduce the review frequency to 1 or less times per week.
ii. Obtain more detailed medical history about current symptoms.
iii. Obtain more detailed medical history and / or history of concomitant diseases, including any pre-existing hepatic disease history or risk factors.
iv. Obtain history of concomitant medications (including OTC medicines, Chinese Traditional Medicines and dietary supplements), alcohol, recreational drugs, and special diets.
v. Exclude acute viral hepatitis caused by hepatitis A, B, C, D, and E virus, hepatotropic virus infection (CMV, EBV, or HSV), autoimmune or alcoholic hepatitis, NASH, hypoxic / ischemic hepatic disease, and biliary disease.
vi. Obtain history of environmental chemical exposure.
vii. Obtain other liver function test results (e.g., INR, DBIL).
viii. Consider a consultation of gastrointestinal or hepatic disease.

12.4 Treatment of hypertension

Angiogenesis inhibitors (e.g., Bevacizumab, Sorafenib) target VEGF pathway, leading to newly-onset hypertension or worsening of previous hypertension. Main mechanisms may include decreasing of NO/PHI2 secreted by endothelial and platelet cells, abnormality of vessel density (small vessels and capillaries) and disturbance of endothelin. Sunitinib may even lead to decreasing of left ventricular ejection fraction (LVEF).

During the first 6 weeks of using angiogenesis inhibitors, subjects should receive weekly blood pressure monitoring. Those who experience hypertension are considered to control blood pressure with following standard treatment: angiotensin II receptor blockers, angiotensin converting enzyme inhibitors, diuretics and adrenaline β-receptor blockers, or combination of listed treatment.

As a reference of management of hypertension caused by angiogenesis inhibitors like Apatinib or Famitinib, hypertension caused by Sorafenib often occurs after 1-2 weeks of treatment and is usually able to control with routine anti-hypertensive treatment. Patients experiencing resistant hypertension are usually in remission by dose modification or discontinuation.

Preferred treatment for hypertension caused by targeted therapy (not metabolized by liver):

i. Valsartan (Diovan): 80-320mg qd
ii. Atenolol: 50-100mg qd
iii. Losartan/Hydrochlorothiazide (Hyzaar): 12.5-100mg qd
iv. Telmisartan (Micardis): 20-80mg qd
v. For resistant hypertension, amlodipine (Norvasc) is preferred: 2.5-10mg qd.

12.5 Treatment of proteinuria

Subjects with urine protein ++ or more twice should receive 24-hour urine protein test.

Subjects will be withdrawn from the study if they experience nephrotic syndrome.

12.6 Prevention and treatment of stomatitis/oral mucositis/mouth ulcers

For prevention the stomatitis, all subjects will be instructed to perform routine “good oral care” each day during the trial. Good oral care will consist of: brushing teeth at least twice daily with soft bristled toothbrush, continue current daily flossing routine (if patients were not already flossing daily, they should not be instructed to start flossing as this could cause oral trauma), and continue routine dental care/maintenance with their dentist, if they have one. It is recommended that patients should use 10mL of an

alcohol-free, 0.5mg/5mL dexamethasone steroid mouthwash swishing and spitting QID, especially during the first 8 weeks of treatment (majority of stomatitis events occur within the first 8 weeks of treatment). The mouthwash is to be held in mouth and swished around mouth to cover the entire buccal mucosa surface for a minimum of two minutes, and then spat out.

Subjects with a clinical history of stomatitis/mucositis/mouth ulcers and those with gastrointestinal morbidity associated with mouth/dental infections, irritation of esophageal mucosa e.g. gastroesophageal reflux disease (GERD) and pre-existing stomatitis/mucositis must be monitored even more closely. Subjects should be instructed to report the first onset of buccal mucosa irritation/reddening to their investigators immediately.

General guidance and management include patient awareness and early intervention. Stomatitis/oral mucositis/mouth ulcers due to everolimus should be treated using local supportive care. Evaluation for herpes virus or fungal infection should be considered. Subjects should be informed about the possibility of developing mouth ulcers/ oral mucositis and instructed to report promptly any signs or symptoms to their investigators. Subjects should be educated about good oral hygiene, instructed to avoid spicy/acidic/salty foods, and should follow the following guidelines:

(i) For mild toxicity (grade 1), use conservative measures such as non-alcoholic mouth wash or normal saline mouth wash several times a day until resolution.

(ii) For more severe toxicity (grade 2 in which case subjects have pain but are able to maintain adequate oral alimentation, or grade 3 in which case subjects cannot maintain adequate oral alimentation), the suggested treatments are topical analgesic mouth treatments (i.e., local anesthetics such as benzocaine, butyl aminobenzoate, tetracaine hydrochloride, menthol, or phenol) with or without topical corticosteroids, such as triamcinolone oral paste 0.1% (Kenalog in Orabase®).

(iii) Agents containing alcohol, hydrogen peroxide, iodine, and thyme derivatives may tend to worsen mouth ulcers. It is preferable to avoid these agents.

(iv)Antifungal agents must be avoided unless a fungal infection is diagnosed. In particular, systemic imidazole antifungal agents (ketoconazole, fluconazole,

itraconazole, etc.) should be avoided in all patients due to their strong inhibition of Everolimus metabolism, therefore leading to higher Everolimus exposures. Therefore, topical antifungal agents are preferred if an infection is diagnosed. Similarly, antiviral agents such as Acyclovir should be avoided unless a viral infection is diagnosed.

12.7 Treatment of non-infectious pneumontis

Non-infectious pneumonitis is a known side effect of rapamycin analogues. Clinically significant pneumonitis is typically accompanied by non-specific symptoms including dyspnea, nonproductive cough, fatigue, and fever. Diagnosis is generally suspected in individuals receiving mTOR inhibitors who develop these symptoms or in asymptomatic individuals in whom a routine chest CT scan reveals a new ground glass or alveolar infiltrate.

The frequency of symptomatic pulmonary toxicity (all grades) was approximately 13% in a phase III study of Everolimus in patients with metastatic renal cell carcinoma. Severe (CTC grade 3) pneumonitis occurred in 4% of patients, and an occasional fatality was reported. The lung toxicity was partly or completely reversible in the majority of cases with interventions including drug interruption, discontinuation and the use of corticosteroids.

Subjects will be routinely questioned as to the presence of new or changed pulmonary symptoms consistent with lung toxicity. CT scans and pulmonary function test should be done, as clinically indicated, if there are symptoms that indicate that the patient has developed non-infectious pneumonitis. If non-infectious pneumonitis develops, the guidelines in Table 5 should be followed. Dose modification instructions are also provided in Table 5. Consultation with a pulmonologist is recommended for any case of pneumonitis that develops during the study.

Table 5 Management of non-infectious pneumonitis and guidelines for dose modification for everolimus

Grade	Required Investigations	Management of Pneumonitis	Dose Modification for Everolimus
Grade 1	CT scans with lung	No specific	Administer 100% of

	windows. Repeat at least every 8 weeks until return to within normal limits.	therapy is required	study treatment dose.
Grade 2	CT scan with lung windows. Consider pulmonary function testing includes: spirometry, DLCO, and room air O2 saturation at rest. Repeat at least every 8 weeks until return to within normal limits. Consider a bronchoscopy with biopsy and / or BAL.	Symptomatic only. Consider corticosteroids if symptoms are troublesome.	Reduce study treatment dose by 1 dose level until recovery to < Grade 1. Study treatment may also be interrupted if symptoms are troublesome. Patients will discontinue study treatment if they fail to recover to < Grade 1 within 28 days.
Grade 3	CT scan with lung windows pulmonary function testing includes: spirometry, DLCO, and room air O2 saturation at rest. Repeat at least every 6 weeks until return to within normal limits. Bronchoscopy with biopsy and / or BAL is	Consider corticosteroids if infective origin is ruled out. Taper as medically indicated.	Hold treatment until recovery to< Grade1. May restart study treatment within 28 days at a reduced dose (by one level) if evidence of clinical benefit.

	recommended.		
Grade 4	CT scan with lung windows and required pulmonary function testing, if possible, includes: spirometry, DLCO, and room air O2 saturation at rest. Repeat at least every 6 weeks until return to within normal limits. Bronchoscopy with biopsy and / or BAL is recommended if possible.	Consider corticosteroids if infective origin is ruled out. Taper as medically indicated.	Discontinuation.

12.8 Management of hyperlipidemia and hyperglycemia

Management of hyperlipidemia should consider previous lifestyle and serum lipids level. Subjects who experience hypercholesterolemia Grade≥2 (>300 mg/dL or 7.75 mmol/L) or hypertriglyceridemia Grade≥2 (>2.5×ULN) should consider receive 3-hydroxy-3-methyl glutaryl (HMG) - coenzyme A reductase (e.g., Atorvastatin, Pravastatin or Fluvastatin) or other appropriate lipid-lowering medication apart from diet control.

Note: Combined treatment of HMG-CoA reductase and Fenofibrate may lead to rare but severe musculoskeletal toxicity, whose symptoms and signs include rhabdomyolysis, significant elevation of CPK, myoglobinuria, acute kidney injury or even death. Thus, risk/benefit ratio for each subject should be calculated before treatment of hyperlipidemia.

There have been reports of hyperglycemia from patients receiving Everolimus. It is suggested to monitor fasting plasma glucose before and after subjects receiving

Everolimus and monitor more frequently if subjects receiving Everolimus with other medication that could lead to hyperglycemia. Optimal glycemic control is suggested before subjects receiving Everolimus.

Table 6 Management of Hyperlipidemia and Hyperglycemia and Guidelines for Dose Modification for Everolimus

Grade	Dose Modification
Grade 1	Continue treatment at investigator discretion and apply symptomatic treatment
Grade 2	Continue treatment at investigator discretion and apply symptomatic treatment
Grade 3	Interrupt until resolved to grade 0-1 and decrease one dose level when resuming treatment
Grade 4	Discontinuation and treatment as appropriate

12.9 Guidelines for supportive care of immune checkpoint inhibitor SHR-1210 or SHR-1316

Subjects will receive appropriate supportive care investigators consider necessary. Supportive care dealing with potential Immune-Related Adverse Effects (irAE) will be listed below, including oral or intravenous corticosteroids, and other anti-inflammatory agents when symptoms are resistant to corticosteroids. Tapering corticosteroids may take several periods because of possible recurring of symptoms. Rule out other possible reasons that may need supportive care, e.g., metastatic diseases or bacterial or viral infection. Supportive treatment will be conducted when investigators ascertain AE is associated with SHR-1210 or SHR-1316 and will not be conducted when AE is not associated with SHR-1210 or SHR-1316.

If capillary endothelial proliferation occurs, conduct biopsy and pathological examination if possible. Subjects experiencing severe and enduring capillary endothelial proliferation are suggested endoscopy and MRI scan to ascertain if there is visceral or mucous involvement.

Symptoms and signs of enterocolitis (e.g., diarrhea, abdominal pain, hematochezia or mucous stool, with or without fever) and intestinal perforation (e.g. peritoneal irritation signs and intestinal obstruction) should be monitored closely. All subjects experiencing diarrhea / colitis will be suggested taking enough liquid. Intravenous infusion of liquid and electrolyte is recommended if oral intake is inadequate. GI consultation and endoscopy are considered to diagnose or rule out colitis for diarrhea Grade ≥2. Oral corticosteroid is considered for diarrhea / colitis Grade 2. Intravenous corticosteroid followed by oral large doses of corticosteroid are considered for diarrhea / colitis Grade ≥3. Taper corticosteroid until recovery to Grade ≤1 and lasts for at least 4 weeks.

For Grade 2 adverse events, use intravenous or oral corticosteroid and monitor liver function more closely until recovery to baseline level (liver function test repeats every week is considered). For Grade 3-4 adverse events, use intravenous corticosteroid for 24-48 hours, and taper corticosteroid until recovery to Grade ≤1 and lasts for at least 4 weeks.

Thyroid disease can occur at any time during treatment, so monitoring subjects for changes in thyroid function (at the beginning of treatment, and at regular intervals during treatment) and clinical signs and symptoms of thyroid disease is necessary. Non-selective adrenaline β-receptor blockers (e.g., propranolol) are recommended as the initial treatment for grade 2 hyperthyroidism. Intravenous corticosteroid followed by oral corticosteroid are recommended for Grade 3-4 hyperthyroidism. Taper until recovery to Grade ≤1 and last at least 4 weeks. Proper hormone replacement therapy may be needed during tapering. Thyroid hormone replacement therapy (e.g. levothyroxine) is considered for Grade 2-4 hypothyroidism.

Intravenous corticosteroid is recommended for Grade 2 pneumonitis. Taper until recovery to Grade ≤1 and last at least 4 weeks. Prophylactic antibiotics should be used in case of long-term use of corticosteroids.

Corticosteroid is recommended for Grade 2 hypophysitis and taper until recovery to Grade ≤1 and last at least 4 weeks. Proper hormone replacement therapy may be needed during tapering. Intravenous corticosteroid followed by oral corticosteroid are

recommended for Grade 3-4 hypophysitis. Taper until recovery to Grade ≤1 and last at least 4 weeks. Proper hormone replacement therapy may be needed during tapering.

Corticosteroid is recommended for Grade 2 adverse events. Intravenous corticosteroid is recommended for Grade 3-4 adverse events. Taper until recovery to Grade ≤1 and last at least 4 weeks.

12.10 Management of infusion related reaction

Infusion reaction may be triggered by infusion of immune checkpoint inhibitor SHR1210 or SHR1316, and it should be graded and managed according to Table 7.

Table 7 Grade and management of infusion reaction

Grade	Symptoms	Treatment	Dose Delay/Modification of SHR1210/SHR1316
Grade 1	Mild transient reaction.	Bedside observation, close monitoring till recovery. Prophylactic medicine shall be given before infusion: Diphenhydramine 50mg, or equivalent and/or Acetaminophen 325-1000mg, administered at least 30mins before the infusion of study medication.	Maintain dose level

Grade 2	Therapy or infusion interruption indicated but responds promptly to symptomatic treatment (e.g., antihistamines, NSAIDS, narcotics, IV fluids). Prophylactic medications indicated for <=24 hours)	Intravenous infusion of normal saline, Diphenhydramine 50 mg IV or equivalent and / or Acetaminophen 325 1000 mg.<br>Bedside observation, close monitoring until the recovery.<br>Corticosteroids could be considered.<br>Record the infusion volume of study drug in CRF.<br>Prophylactic medicine shall be given before infusion: Diphenhydramine 50mg, or equivalent and/or Acetaminophen 325 1000mg, administered at least 30mins before the infusion of study medication. Glucocorticoid (equal to Hydrocortisone 25mg) if necessary.	Interruption.<br>Re-administration after symptoms disappear at 50% of the initial infusion rate. If there is no complication within 30 minutes, increase to the original 100% infusion rate. Closely monitor. If symptoms recur, discontinue the drug.

Grade ≥3	Grade 3: Prolonged (e.g., not rapidly responsive to symptomatic medication and / or brief interruption of infusion). Recurrence of symptoms following initial improvement. Hospitalization indicated for clinical sequelae. Grade 4: Life-threatening consequences. Urgent intervention indicated.	The infusion of study medication shall be immediately discontinued. Recommend bronchodilator, subcutaneous Epinephrine 0.2-1mg 1: 1000 solution, or 0.1-0.25mg 1: 10000 solution slow iv injection, if necessary, and / or intravenous injection equivalent to Diphenhydramine 50 mg + 100 mg Methylprednisolone. Comply with guidelines for allergic reactions of the study site. Bedside observation, close monitoring till recovery.	Discontinuation

13 Management of Complimentary Trial Compounds

13.1 Dispensation of compounds

This research is conducted in department of breast surgery, Fudan university Shanghai Cancer Center. Enrolled subjects may acquire compounds from Hengrui Medicine according to Table 1 which are under unified management of the hospital, obtain medication number and corresponding study medication will be distributed. Designated personnel of the hospital is put in charge of keeping compounds, fill in records of receiving and using compounds and retrieve the remaining compounds and empty bottles promptly during the test. The usage and records of compounds should be checked at regular intervals and deal with retrieved medication at any time.

13.2 Storage and Management of Compounds

According to Good Clinical Practice (GCP), study medication is uniformly stored, distributed and recycled by the study site. Compounds are stored sealed, protected from light at room temperature, with a tentative expiration date of 2 years.

13.3 Disposal of Remaining Compounds

Investigators should record the date and dosage of each subject's medication. The total amount of study medication is 120% of the pre-designed dosage. The remaining compounds should be returned to Hengrui Medicine at the end of the trial.

14 Adverse Events

14.1 Definition

An adverse event (AE) is any undesirable medical event experienced by clinical trial subjects after administration of certain compounds, however, AE does not always reflect a causal relationship with intervention.

14.2 Adverse event reporting period

The reporting period starts from enrollment and lasts till the final follow up. Any adverse event happens during this period should be filled in the case report form.

14.3 Serious adverse event (SAE)

An adverse event is defined as a SAE if it agrees with one or more following

criteria:

i. Death.
ii. Life-threatening.
iii. Hospitalization (initial or prolonged).
iv. Disability or Permanent Damage.
v. Congenital Anomaly/Birth Defect, important Medical Events.

14.4 Report of severe adverse event

For all serious adverse events the trial should be suspended immediately and corresponding measures to protect subjects should be practiced. Serious adverse events will be recorded in a table and reported to the director of the unit and the sponsor within 24 hours by phone or fax. The investigator shall fill in the severe adverse event report form and fax it to State Food and Drug Administration. Investigator should follow the severe adverse event till resolution. Relevant medical documents should be achieved within original material, including report sheet of laboratory test (e.g. X-ray examination, electrocardiogram, etc.).

14.5 Record and report

The investigator should explain to the patient in detail and ask the patient to truthfully reflect the change in the condition after the administration. Physicians should avoid induced question. While observing the curative effect, the physicians pay close attention to observing adverse events, analyzing the causes, making judgments, and following up observations and records to count the incidence of adverse reactions.

For adverse event occurred during the trial, its time of onset, symptoms, severity, duration, treatment and prognosis should be recorded in the case report, so as to evaluate its significance with test compound. The detailed record should be provided, signed and dated by investigators. The adverse events will be graded based on NCI-CTC 3.0. For each symptom, the highest grade experienced since last follow-up should be reported.

Determination of relationship between adverse events and clinical trial: Attribute to one of the five categories: definitely related, probably related, probably unrelated, unrelated and not appreciable. The first two categories are considered as adverse event and the proportions of adverse events will be calculated.

i. Definite attribution: The chronological order of adverse event after trial onset is

reasonable and the reaction is consistent with the known type of reaction. The situation improves after dis-administration and the reaction reappears after re-administration

ii. Probably related: The chronological order of adverse event after trial onset is reasonable and the reaction is consistent with the known type of reaction. the clinical condition or alternative intervention may also cause such reaction.

iii. Probably unrelated: The chronological order of adverse event after trial onset is less reasonable and the reaction is less consistent with the known type of reaction, the clinical condition or alternative intervention may also cause such reaction.

iv. Unrelated: The chronological order of adverse event after trial onset is unreasonable and the reaction is inconsistent with the known type of reaction, the clinical condition or alternative intervention may also cause such reaction. Improvement of disease status or stop intervention other than test compounds lead to elimination of such reaction, which will relapse with the restart of alternative intervention.

v. Not appreciable: The onset of reaction and trial lack clear chronological order and the reaction is similar with known reaction type. Concurrent use of other medication may cause same reaction.

Causality categories between adverse events and test compound

	Definite attribution	Probably related	Possibly related	Conditional related	Unrelated
Event or laboratory test abnormality, with plausible time relationship to compounds intake.	+	+	+	+	—
Event definitive pharmacologically or phenomenologically.	+	+	+	—	—
Response to withdrawal plausible.	+	+	±	±	—
Rechallenge satisfactory, if necessary	+	?	?	?	—
Cannot be explained by disease or other compounds	+	+	—	±	—

15 Data Processing

15.1 Requirements of data filled by investigators

For all the subjects who have filled in the informed consent and selected to enter the trial, every item in the case report form should be recorded carefully and in detail, and no blank or missing item is allowed (the blank space without record should be crossed).

i. All data in the case report form should be checked with the subject's medical record data to ensure accuracy.
ii. As the original data, the case report form is only allowed to be crossed with any correction made, and the corrected data should be annotated with the signature of the investigator and the date.
iii. The copy of the laboratory test reports should be placed after the case report form.
iv. Data that are significantly higher or beyond the clinical acceptance range should be verified and necessary explanation should be made to subjects by the investigator.
v. Please refer to the case report form for instructions.

15.2 Data traceability and completion of CRF

The original record is the study medical record for proper preservation. The case report form from the research medical record is filled in by the researcher. Each included case must complete the case report form.

16 Data Set for Statistical Analysis

After the completion of the trial scheme and case report form, the analysis plan shall be formulated, and necessary modifications shall be made during the trial process. The plan shall be completed before data locking, and the statistical analysis report will be provided after data analysis.

16.1 Analysis data set

i. Full Analysis Set (FAS): Full analysis set refers to the collection of qualified cases and shedding cases, with the exception of the cases excluded.
ii. Per-Protocol population (PP) Data Set: The PP data set is defined restricted to all

the cases that meet the inclusion criteria and complete the treatment plan, with good compliance, no banned compounds and fulfilled required contents in case report form, as well as the observation record documents from subjects whose compliance satisfies the study protocol requirement.

iii. Safety Data Set: Safety Data Set refers to the data with safety records after receiving at least one treatment, with the exception of the cases excluded.

16.2 Method of statistics

i. Whether subject to normal distribution: if not, modify statistical methods or perform data transformation.

ii. Whether there is outlier: make statistical and professional analysis, and decide whether to include or not.

iii. Whether there is missing value: when a primary therapeutic index of individual subject fails to be measured, the last observation data should be transferred.

iv. The percentage of dropouts should not exceed 20%, otherwise it requires analysis and explanation.

v. Descriptive statistical analysis: e.g., mean, standard deviation, maximum, minimum, confidence interval, rate, etc.

16.3 Method of analysis

i. Measurement data: Use t test, paired t test, rank sum test, paired rank sum test, etc.

ii. Enumeration data: Use Fisher's exact test, etc., rank sum test is adopted to ranked data.

iii. Analysis of efficacy indicators: CMH test, chi square test or logistic regression will be used for enumeration data. Analysis of variance or rank sum test will be used for measurement data according to the feature of the data. Kaplan Meier method or Cox regression will be used for survival data.

iv. FAS analysis and PP analysis: PP analysis and FAS analysis will be conducted simultaneously for the main efficacy indicators.

16.4 Statistic expressions

i. The report is mainly represented by tables with title, annotation and number of cases, which are self-evident.

ii. Two-sided P values will be calculated for all statistical tests. A value of $P < 0.05$ is considered significant.

16.5 Analysis software

All statistical analyses will be performed using R version 3.6.1 (Foundation for Statistical Computing, Vienna, Austria).

16.6 Interim Analysis

An interim analysis will be conducted when 20 subjects are enrolled in at least one arm, and at least one subject is enrolled in each arm, to preliminarily evaluate the efficacy and safety of the drug combination in each arm. With the estimated enrollment speed, around 50% of subjects would have been enrolled by the interim analysis time point.

17 Quality Control and Quality Assurance

Regular supervision and inspection will be performed during the trial to ensure the implementation of study protocol. The raw data will be reviewed to ensure the consistency with data in the case report form.

18 Ethical Principle

The study procedure must strictly conform to the requirement of Good Clinical Practice of SFDA and Declaration of Helsinki.

Institutional Ethics Committee (IEC)

This protocol and written informed consent as well as material directly related with subjects should be submitted to ethics committee. The trial can only initiate after achievement of written approval of the ethics committee.

Informed consent form (ICF)

Prior to enrollment the investigators are responsible for oral and written consent about information including objective, procedure and potential risks of the study to every subject. The subject should be informed about the right to decide whether to participate in the trial and that subject is free to withdraw from trial any time willingly. Subjects or their legal representatives will read and understand the informed consent form and sign it, and keep the copy of signature page.

19 Trials Progress and Data Retention.

19.1 Trials progress

Duration of inclusion:30 months (From October 2018 to February 2021).

Duration of follow-up after treatment:12 months after inclusion of the last subject.

Date of primary analysis: February 2021.

19.2 Data retention

The case report forms will be confirmed with signature by investigators. After completion of trial, all case report forms, detailed materials about classic cases and clinical trial record forms will be conserved. Investigators will keep original materials relevant to subjects of CR or PR, laboratory results, signed informed consent originals and copy of case report forms.

20 References

1. Perou CM, Sorlie T, Eisen MB, et al. Molecular portraits of human breast tumours. Nature. 2000; 406:747-52.
2. Sørlie T, Perou CM, Tibshirani R, et al. Gene expression patterns of breast carcinomas distinguish tumor subclasses with clinical implications. Proc Natl Acad Sci U S A. 2001; 98:10869-74.
3. Foulkes WD, Smith IE, Reis-Filho JS. TNBC. N Engl J Med. 2010; 363: 1938-48.
4. Carey L, Winer E, Viale G, et al. TNBC: disease entity or title of convenience? Nat Rev Clin Oncol. 2010; 7: 683-92.
5. Metzger-Filho O, Tutt A, de Azambuja E, et al. Dissecting the heterogeneity of TNBC. J Clin Oncol. 2012; 30:1879-87.
6. Shah SP, Roth A, Goya R, et al. The clonal and mutational evolution spectrum of primary TNBCs. Nature. 2012; 486:395-9.
7. Burstein MD, Tsimelzon A, Poage GM, et al. Comprehensive genomic analysis identifies novel subtypes and targets of TNBC. Clin Cancer Res. 2015;21:1688-1698.
8. O'Shaughnessy J, Schwartzberg L, Danso MA, et al. Phase III study of iniparib plus gemcitabine and carboplatin versus gemcitabine and carboplatinin patients with metastatic TNBC. J Clin Oncol. 2014;32(34):3840-7.
9. Tutt A, Ellis P, Kilburn L, et al. TNT: A rand omized phase III trial of Carboplatin (C) compared with Docetaxel (D) for patients with metastatic or recurrent locally advanced triple negative or BRCA1/2 breast cancer (CRUK/07/012). Cancer Res, 2015, 75(9 Suppl): Abstr S3-01.
10. Tutt A, Tovey H, Cheang MCU, et al. Carboplatin in BRCA1/2-mutated and TNBC BRCAness subgroups: the TNT Trial. Nat Med. 2018;24(5):628-637.
11. Lehmann BD, Bauer JA, Chen X, et al. Identification of human TNBC subtypes and preclinical models for selection of targeted therapies. J Clin Invest. 2011;121:2750-2767.
12. Lehmann BD, Jovanović B, Chen X,et al. Refinement of TNBC Molecular Subtypes: Implications for Neoadjuvant Chemotherapy Selection. PLoS One. 2016 Jun 16;11:e0157368.

13. Burstein MD, Tsimelzon A, Poage GM, et al. Comprehensive genomic analysis identifies novel subtypes and targets of TNBC. Clin Cancer Res. 2015;21:1688-1698.
14. Jiang YZ, Ma D, Suo C, et al. Genomic and Transcriptomic Landscape of TNBCs: Subtypes and Treatment Strategies. Cancer Cell, 2019 Mar 18; 35(3):428-440.
15. Liu YR, Jiang YZ, Xu XE, et al. Comprehensive transcriptome analysis identifies novel molecular subtypes and subtype-specific RNAs of TNBC. Breast Cancer Res. 2016;18:33.
16. Denkert C, von Minckwitz G, Brase JC, et al. Tumor-infiltrating lymphocytes and response to neoadjuvant chemotherapy with or without carboplatin in human epidermal growth factor receptor 2-positive and triple-negative primary breast cancers. J Clin Oncol. 2015;33:983-991.
17. Ibrahim EM, Al-Foheidi ME, Al-Mansour MM, et al. The prognostic value of tumor-infiltrating lymphocytes in TNBC: a meta-analysis. Breast Cancer Res Treat. 2014;148:467-476.
18. Robson M, Im SA, Senkus E, et al. Olaparib for Metastatic Breast Cancer in Patients with a Germline BRCA Mutation. N Engl J Med. 2017;377(6):523-533.
19. Ma F, Li Q, Chen S, et al. Phase I Study and Biomarker Analysis of Pyrotinib, a Novel Irreversible PanErbB ReceptorTyrosine Kinase Inhibitor,in Patients With Human Epi dermal Growth Factor Receptor 2-Positive Metastatic Breast Cancer. J Clin Oncol. 2017;35(27):3105-3112.
20. Xu B et al. Phase II Study of Pyrotinib Plus Capecitabine Versus Lapatinib Plus Capecitabine in Patients With HER2+Metastatic Breast Cancer. San Antonio Breast Cancer Symposium, December 5-9, 2017. PD3-08

Precision Treatment of Refractory Triple Negative Breast Cancer Based on Molecular Subtyping

(FUSCC-TNBC- umbrella)

FUTURE Trial

Statistical Analysis Plan (SAP)

Study institute: Fudan University Shanghai Cancer Center

Major study investigator: Zhi-Ming Shao, MD. PhD.

ClinicalTrials.gov Identifier: NCT 03805399

Contents

1. **Introduction** 65
1.1. **Study Design** 65
1.2. **Sample Size** 65
2. **Study Endpoints** 65
2.1. **Efficacy Endpoints** 65
2.1.1. **Objective response rate, ORR** 65
2.1.2. **Disease Control Rate, DCR** 65
2.1.3. **Progression Free Survival, PFS** 65
2.1.4. **Overall Survival, OS** 66
2.1.5. **Other Analyses Related to Efficacy** 66
2.2. **Safety Endpoints** 67
3. **Statistical Analysis** 67
3.1. **General Considerations** 67
3.1.1. **Analysis Sets** 67
3.1.2. **Methods for Handling Missing Data** 68
3.2. **Study Subjects** 71
3.2.1. **Subjects Disposition** 71
3.2.2. **Demographics** 71
3.2.3. **Medical History** 71
3.2.4. **Major Protocol Deviations** 71
3.3. **Efficacy Analysis** 72
3.4. **Safety Analysis** 72
3.4.1. **Extent of Exposure** 72
3.4.2. **Adverse Events** 73
3.4.3. **Laboratory Evaluations** 73
3.4.4. **Vital Signs** 74
4. **Interim Analysis** 74

1. Introduction

The FUTURE trial is a phase Ib/II biopsy-mandated, subtyping-based and genomic biomarker-guided umbrella trial to evaluate if targeting the subtypes therapeutically might improve patient outcomes.

1.1 Study Design

This is a Phase Ib/II, open-label, umbrella study.

1.2 Sample Size

Based on the different multi-gene expression profiles and the potential molecular characteristics of different pathways, seven treatment arms and six treatment groups were initially set up to eroll 20 patients per treatment arm. Therefore, a total of 140 patients were enrolled in this study. 3 or more than 3 of 20 patients in each arm group reached CR or PR, will be defined to reach the study end point.

2. Study Endpoints

2.1 Efficacy Endpoints

2.1.1 Objective response rate, ORR

The primary endpoint will be the objective response rate (ORR) [PR+CR], with responders requiring a confirmatory response assessment no sooner than 4 weeks after the first responseassessment.Assessment of tumor response is based upon on-site readings by local radiologists,using RECIST1.1.

2.1.2 Disease Control Rate, DCR

One of the secondary endpoints will be disease control rate(DCR) [CR+PR+SD].

2.1.3 Progression Free Survival, PFS

One of the secondary endpoints will be progression free survival (PFS). Progression-free survival is defined as the interval from the first dose start date to the date of disease progression defined as documented PD or death from any cause, whichever occurs first.

2.1.4 Overall Survival, OS

Overall survival is defined as the time fromthe date of the first dose start date to the date of death due to any cause. Patients without documentation of death at the time of the data cut off for analysis will be censored at the date the patient was last known to be alive or the data cut off date,whichever is earlier. The last known alive date is the last record in the study database. This date may be the maximum of the last visit date or last contact date, including telephone follow-up where the patientis known to be alive.

2.1.5 Other Analyses Related to Efficacy

Duration of response (DOR) will be calculated as the date of the first evaluation showing documented PR or CR to the date of the first PD or death, whichever is earlier.

Swimmer plots of treatment duration showing the date of progression or death, whichever is earlier, will be presented. Treatment ongoing status will be marked at the end of the plot. Waterfall plots of the percent change from baseline in target lesion measurement will be presented.

Example of swimmer plot:

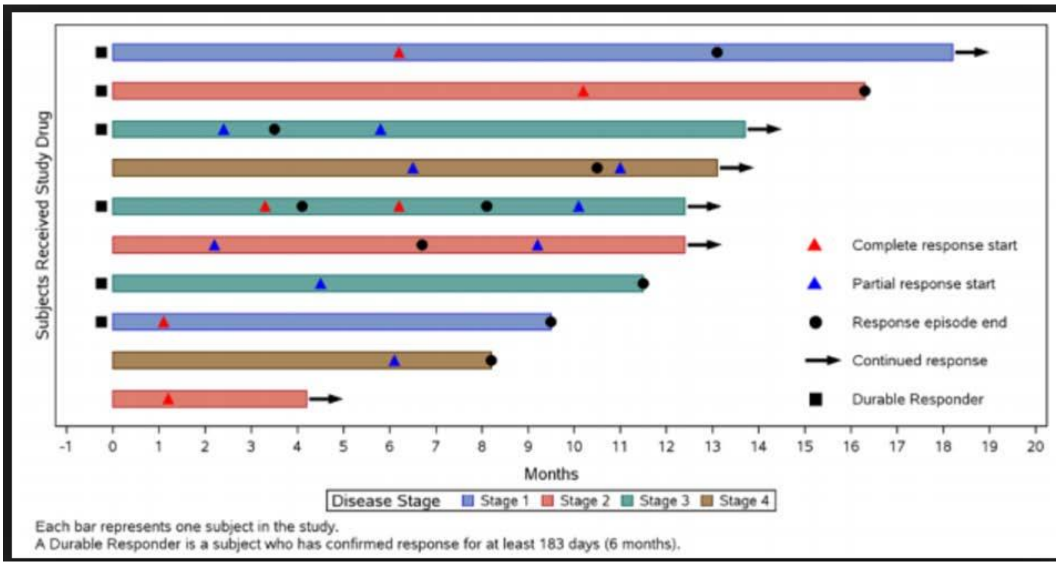

Example of waterfall plot:

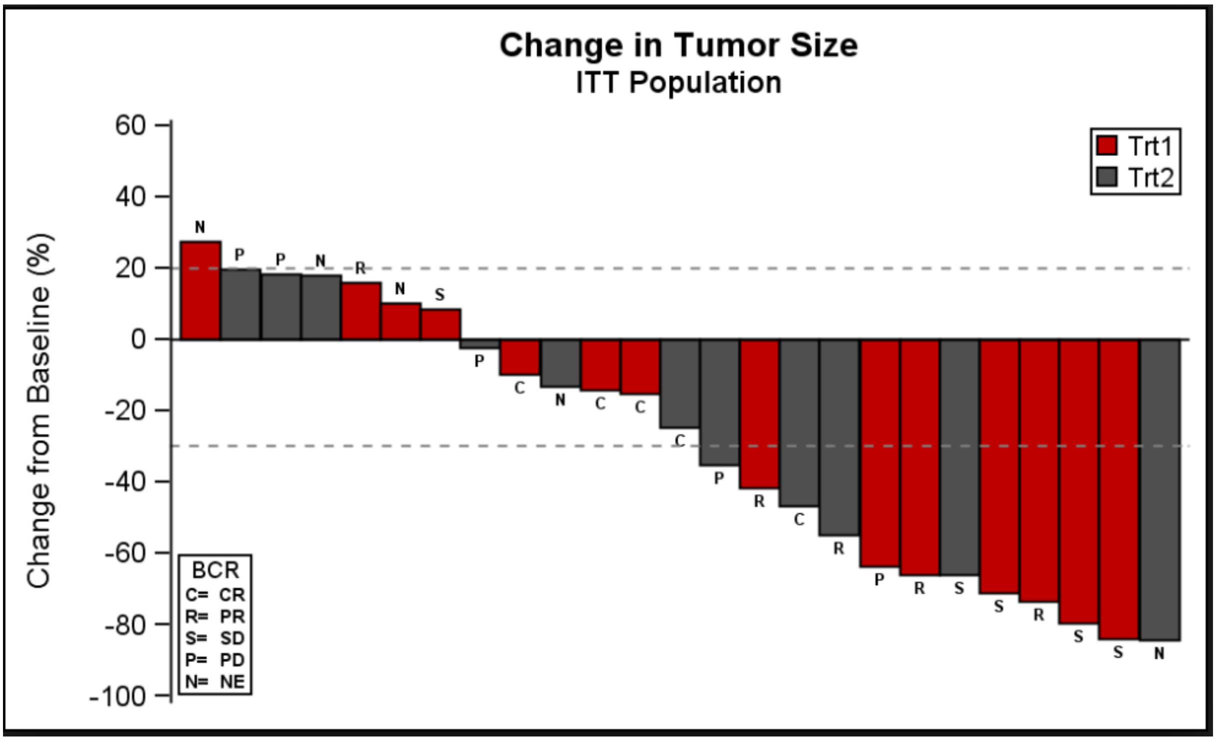

2.2 Safety Endpoints

Safety will be assessed for overall safety population. Data will be presented in terms of AEs, laboratory data, and vital signs.

- Adverse Events
- Clinical Laboratory Evaluations
- Vital Signs

3. Statistical Analysis

All analyses were based on descriptive statistics, without formal statistical hypothesis testing. As mentioned above, 3 or more than 3 of 20 patients in each arm group reached CR or PR, will be defined to reach the study end point.

3.1 General Considerations

3.1.1 Analysis Sets

Per-Protocl (PP) Set: The PP data set is defined restricted to all the cases that meet the inclusion criteria and complete the treatment plan, with good compliance, no banned compounds and fulfilled required contents in case report form, as well as the

observation record documents from subjects whose compliance satisfies the study protocol requirement.

Intention to Treat (ITT) Set: The ITT population includes all patients who have signed informed consent, regardless of their adherence with the entry criteria, regardless of the treatment they actually received, and regardless of subsequent withdrawal from treatment or deviation from the protocol.

Safety Set: This will include all patients who received at least 1 dose of medication, irrespective of dose.

3.1.2 Methods for Handling Missing Data

Missing or partial dates will not be imputed except for AE and concomitant medication data. In this case the listings will show these dates as missing, but the following approach will be used to define whether an AE is treatment-emergent or a therapy is considered a prior medication.

Missing Data Imputation for Adverse Event/Concomitant Medication Start Dates

If the stop date is non-missing and the imputed start date is after the stop date, the stop date will be used as the start date.

(1) Missing day only

• If the month and year of the AE/the concomitant medication are the same as the month and year of the first dose date, the first dose date will be used.

• If the month and year are before the month and year of the first dose date, the last day of the month will be assigned to the missing day.

• If the month and year are after the month and year of the first dose date, the first day of the month will be assigned to the missing day.

(2) Missing day and month

• If the year is the same as the year of the first dose date, the first dose date will be used.

• If the year is prior to the year of the first dose date, December 31st will be assigned to the missing fields.

• If the year is after the year of the first dose date, January 1st will be assigned to the missing fields.

(3) Missing day, month, and year

• The first dose date will be used.

Missing Data Imputation for Missing Adverse Event/Concomitant Medication Stop Date

If the start date is non-missing and the imputed stop date is before the start date, the start date will be used. If the death date is available and the imputed stop date is after the death date, the death date will be used.

(1) Missing day only

• The last day of the month will be assigned as the missing day.

(2) Missing day and month

• December 31st will be assigned to the missing fields.

(3) Missing day, month and year

• The event will be regarded as ongoing.

Missing/Partial Dates during Screening Visit

The following rules apply to dates recorded during the screening visits (eg, prior therapies/medications, medical history):

(1) Missing day only

• The first day of the month will be used if the year and the month are the same as those for the first dose of study drug. Otherwise, the 15th will be used.

(2) Missing day and month

• If the year is the same as the year of the first dose of study drug, the 15th of January will be used unless it is later than the first dose, in which case the date of the first of January will be used.

• If the year is not the same as the year of the first dose of study drug, the 15th of June will be used, unless other data indicates that the date is earlier.

(3) Missing day, month, and year

• No imputation will be applied.

Missing Last Dosing Date

Missing/incomplete last dose date from the treatment discontinuation page will be imputed as follows:

(1) Missing day only

• If the treatment discontinuation reason is death, the death date will be used.

• Else if the last available dosing date from dosing data matches the partial last dose date from the treatment discontinuation page, the last available dosing date will be used.

• Else the first day of the month will be used.

(2) Missing day and month

• If the treatment discontinuation reason is death, the death date will be used.

• Else if the last available dosing date from dosing data matches the partial last dose date from the treatment discontinuation page, the last available dosing date will be used.

• Else January 1 will be used.

(3) Missing day, month and year

• If the treatment discontinuation reason is death, the death date will be used.

• Else the last available dosing date will be used.

The imputed last dose date will be compared to the available study discontinuation date and the data cutoff date. Then the earliest date will be used.

3.2 Study Subjects

3.2.1 Subjects Disposition

Disposition in terms of number of patients screened/entered into the study, treated, permanently discontinued treatment, and reasons for treatment discontinuation will be summarized for the ITT Population and the Safety Population. The number of patients included in each population will be summarized by way of a flow chart. A by-patient listing for disposition will be provided, including whether the patient is included in each of the analysis sets, treatment status, date of stopping treatment, date of stopping study participation, reason for treatment discontinuation, study completion status, and survival follow-up status.

A separate listing will be provided for patients who were registered into the study but did not receive study drug and the reason for not receiving study drug. Screen failures and entered-butnot-dosed patients will be excluded from all analyses.

3.2.2 Demographics

Baseline characteristics will include age, Eastern Cooperative Oncology Group (ECOG) performance status, numbers of metastatic organs and metastaic sites at initial diagnosis and at screening.

3.2.3 Medical History

Prior systemic anticancer therapy will be summarized. Number of prior anticancer therapies for metastatic disease (＜3 prior lines vs 3-6 prior lines vs ＞6 prior lines) and types of prior chemotherapies for metastatic disease will also be summarized categorically.

3.2.4 Major Protocol Deviations

A by-patient listing with major study protocol violations and deviations will be provided for patients.

<u>Protocol Violations to be Programmed</u>

- Inclusion/Exclusion Criteria

- <2 weeks between previous treatment, immune therapy, chemotherapy, or investigational therapy for metastatic disease and start of treatment.

- Informed Consent

- Patient's written informed consent not available.

- Patient's written informed consent too late (after start of study-specific procedures)

- Prohibited Medication

- Anticancer therapy during treatment.

- Radiation during treatment.

- Prophylactic medication of hematopoietic growth factors or blood transfusions before Cycle 1.

3.3 Efficacy Analysis

Formal statistical hypothesis-testing will not be performed. Descriptive statistics, data summaries and graphical methods will be used to assess the efficacy. The primary endpoint will be the objective response rate (ORR) [PR+CR], with responders requiring a confirmatory response assessment no sooner than 4 weeks after the first response assessment. Assessment of tumor response is based upon on-site readings by local radiologists, using RECIST 1.1. Earliest imaging date of associated imaging methods was used as the response assessment date. Secondary efficacy endpoints will include disease control rate (DCR, CR+PR+SD), progression-free survival (PFS), and overall survival (OS). PFS, and OS data will be analyzed via Kaplan-Meier method and 95% CI from Clopper-Pearson method with log-log transformation.

3.4 Safety Analysis

Safety will be assessed for Safety Population. Data will be presented in terms of AEs, laboratory data, and vital signs.

3.4.1 Extent of Exposure

The extent of exposure mainly summarizes the drug exposure time, the cumulative dosage and the drug intensity.

3.4.2 Adverse Events

Treatment-emergent adverse events (TEAEs) are defined as any AEs that begin or worsen on or after the start of study drug through 30 days after the last dose of study drug. All AEs will be coded using Medical Dictionary for Regulatory Activities (MedDRA) Version 20.0 unless otherwise specified. The severity will be graded based on the National Cancer Institute's (NCI) Common Terminology Criteria for Adverse Events (CTCAE) Version 4.03. All AEs will be listed. Only TEAEs will be summarized and will be referred to as AEs hereafter. Timing of AEs and concomitant medications will take account of the date and the time of the AE or concomitant medication. The frequency and severity of AEs will be tabulated by MedDRA SOC and PT. For this purpose, an AE that occurs more than once within each patient will be counted only once (at the worst CTCAE grade and relationship category). Additional by-patient listings will be provided for AEs leading to on-treatment death, serious AEs (SAEs), and AEs leading to discontinuation of treatment (excluding AEs leading to death).

3.4.3 Laboratory Evaluations

Clinical laboratory data results will be reported in standard international units and Chinese conventional units. Baseline is defined as the last observation occurring prior to the first treatment administration of medication. Observations occurring on the same day as first treatment administration may be the baseline assessment only if the time of assessment occurs prior to the time of treatment. If this cannot be determined, the observation will be assumed to have occurred after dosing. If a lab value is reported using a non-numeric qualifier (eg, less than [<] a certain value, or greater than [>] a certain value), the given numeric value will be used in the summary statistics, ignoring the non-numeric qualifier. Hematology and serum chemistry data will be listed by patient and summarized by study visit. Actual values by visit and change-from-baseline will be summarized by mean, median, standard deviation, minimum, maximum and number of patients. Shift tables from baseline to worst CTCAE grade on treatment and from worst to last CTCAE grade on treatment will be presented where CTCAE grade is available. Shift tables will be presented based on CTCAE v4.03 criteria, using the grades 1 through 4 as well as a grade 0 indicating no abnormality. These shift tables

will report the shift from baseline CTCAE grade to worst grade on treatment and from worst to last on-treatment visit

3.4.4 Vital Signs

The actual value and change from baseline (most recent evaluation within 28 days prior to beginning study therapy) to each on-study evaluation, including baseline and end of treatment, will be summarized for vital signs. Vital sign measurements will be presented for each patient in a by-patient data listing.

4. Interim Analysis

An interim analysis will be conducted when 20 subjects are enrolled in at least one arm, and at least one subject is enrolled in each arm, to preliminarily evaluate the efficacy and safety of the drug combination in each arm. With the estimated enrollment speed, around 50% of subjects would have been enrolled by the interim analysis time point.
