# Supplementary material for: Molecular subtyping and genomic profiling expand precision medicine in refractory metastatic triple-negative breast cancer: the FUTURE trial
Source: Cell Res. 2020 Jul 27;31(2):178–86. doi: 10.1038/s41422-020-0375-9 (PMC8027015; doi:10.1038/s41422-020-0375-9)
Supplement: Supplementary file 2 — Supplementary information, Fig. S1 [file 41422_2020_375_MOESM2_ESM.pdf]

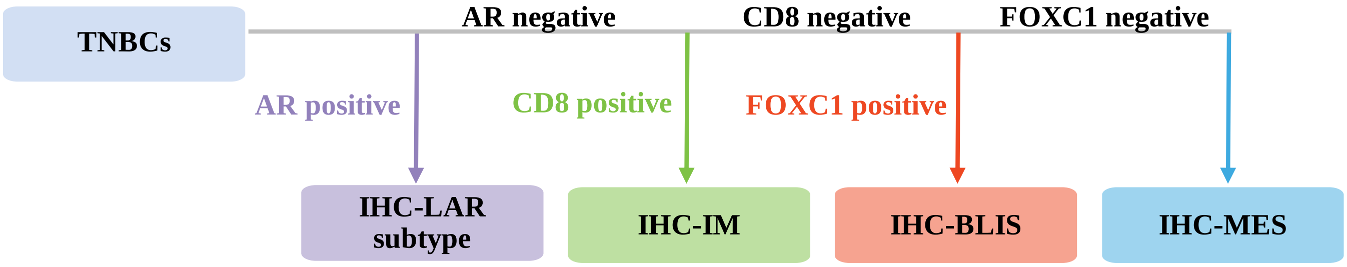

Figure S1. Immunohistochemistry (IHC) based method defines the four subtypes of triple-negative breast cancer (TNBC)
Abbreviations: LAR, luminal androgen receptor; IM, immunomodulatory; BLIS, basal-like immune-suppressed; MES, mesenchymal-like.
