# Supplementary figures and images for: Molecular subtyping and genomic profiling expand precision medicine in refractory metastatic triple-negative breast cancer: the FUTURE trial
Source: Cell Res. 2020 Jul 27;31(2):178–86. doi: 10.1038/s41422-020-0375-9 (PMC8027015; doi:10.1038/s41422-020-0375-9)

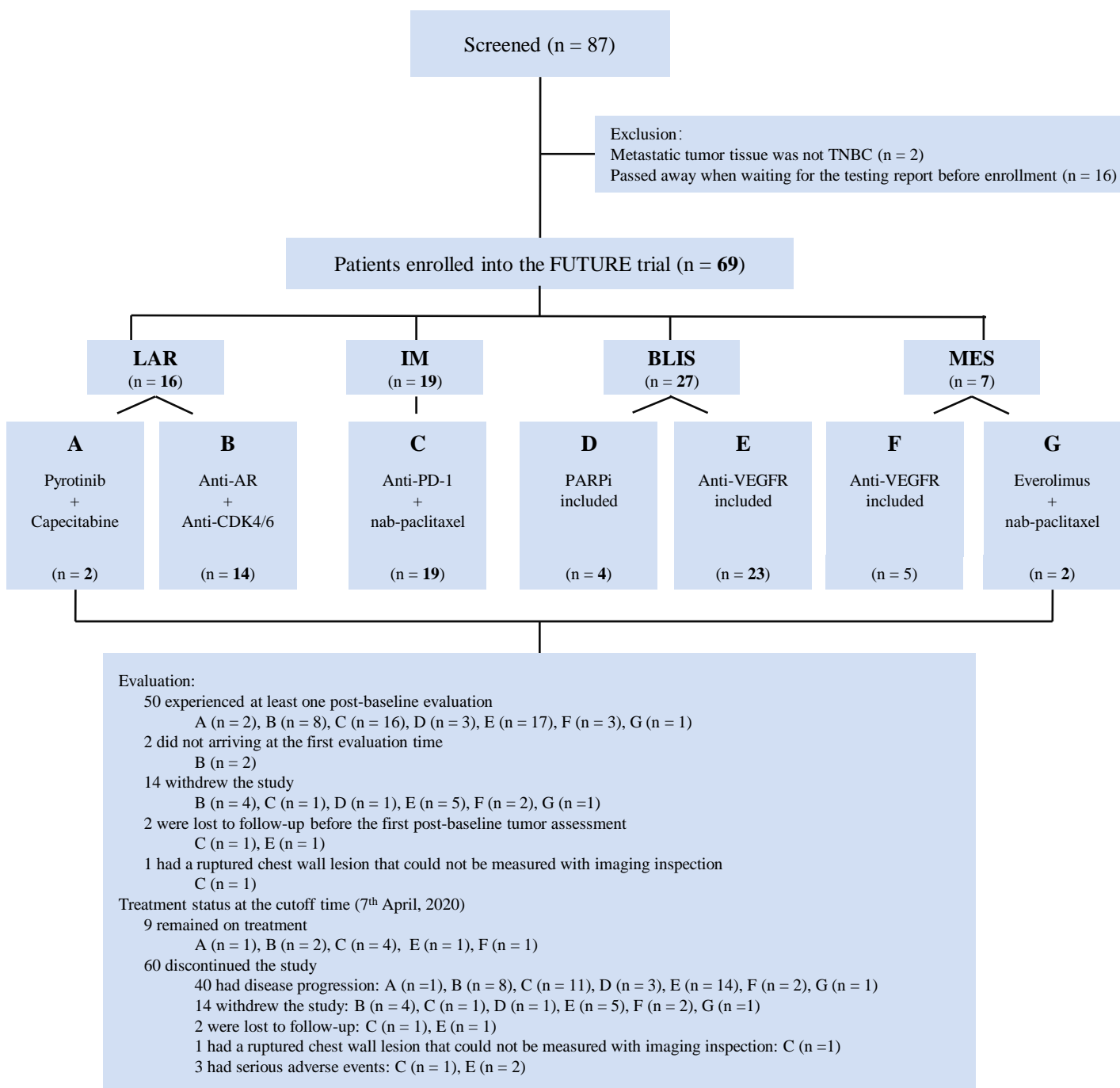

**Figure S2. The flow diagram of patient enrollment and treatment assignments**

Supplement: Supplementary file 3 — Supplementary information, Fig. S2 [file 41422_2020_375_MOESM3_ESM.pdf]

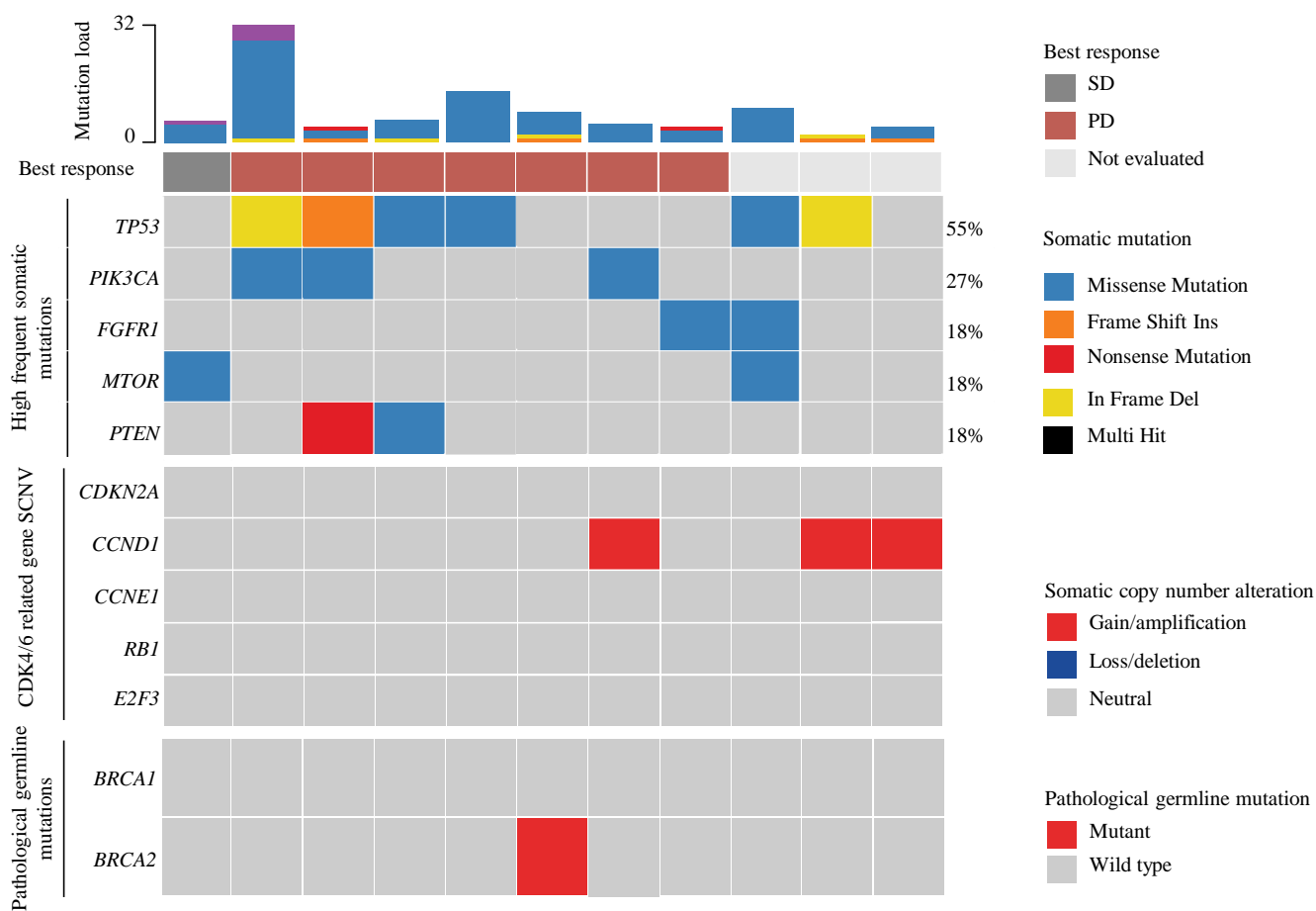

**Figure S5. Genomic landscape of TNBCs in arm B**

Supplement: Supplementary file 6 — Supplementary information, Fig. S5 [file 41422_2020_375_MOESM6_ESM.pdf]
