# Supplementary material for: Molecular subtyping and genomic profiling expand precision medicine in refractory metastatic triple-negative breast cancer: the FUTURE trial
Source: Cell Res. 2020 Jul 27;31(2):178–86. doi: 10.1038/s41422-020-0375-9 (PMC8027015; doi:10.1038/s41422-020-0375-9)
Supplement: Supplementary file 4 — Supplementary information, Fig. S3 [file 41422_2020_375_MOESM4_ESM.pdf]

Figure S3. Representative samples, and their images in the FUTURE trial

Abbreviations: CR, complete response; PR, partial response; SD, stable disease; PD, progressive disease.

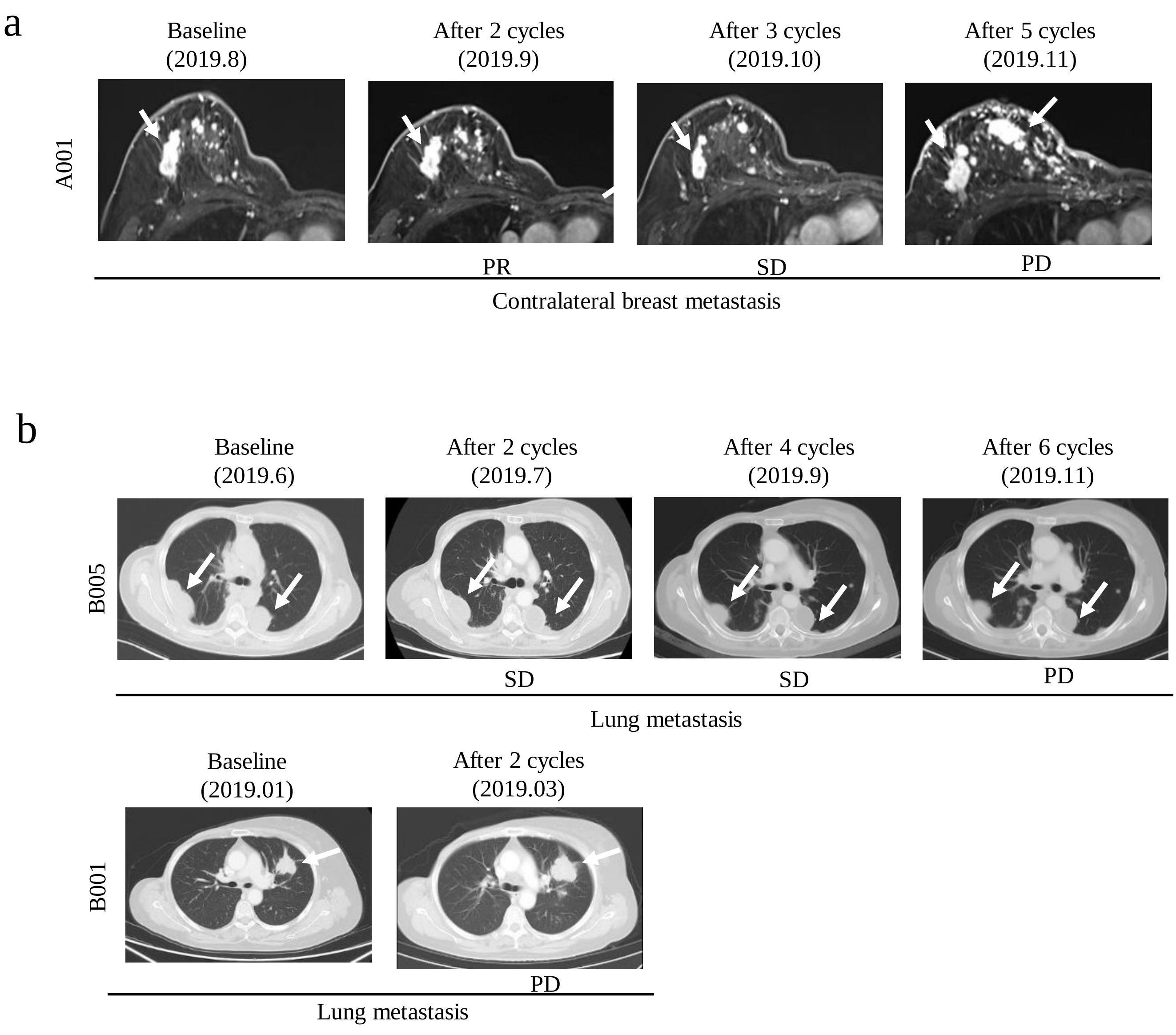

c

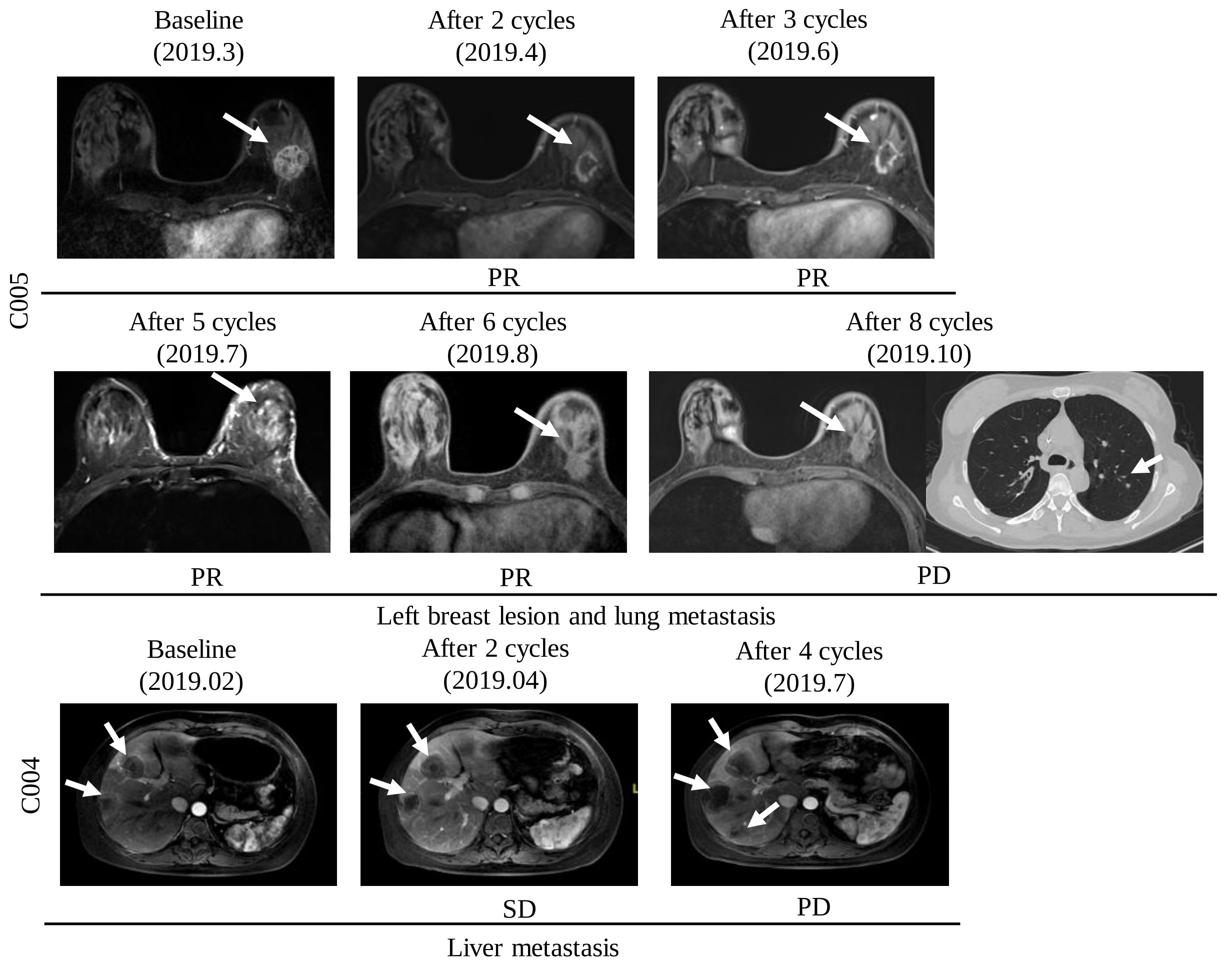
C005
Baseline
(2019.3)
After 2 cycles
(2019.4)
After 3 cycles
(2019.6)
PR
PR
After 5 cycles
(2019.7)
After 6 cycles
(2019.8)
After 8 cycles
(2019.10)
PR
PR
PD
Left breast lesion and lung metastasis
C004
Baseline
(2019.02)
After 2 cycles
(2019.04)
After 4 cycles
(2019.7)
SD
PD
Liver metastasis

d

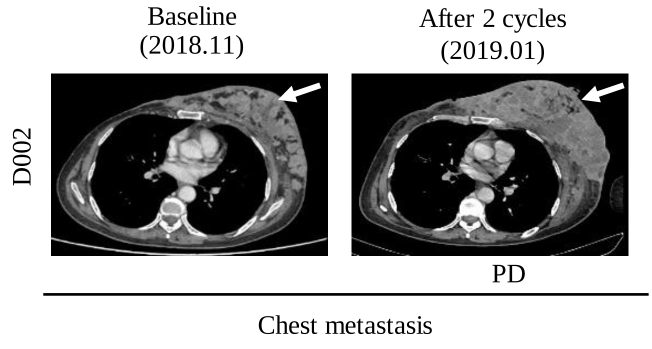
D002
Baseline
(2018.11)
After 2 cycles
(2019.01)
PD
Chest metastasis

e

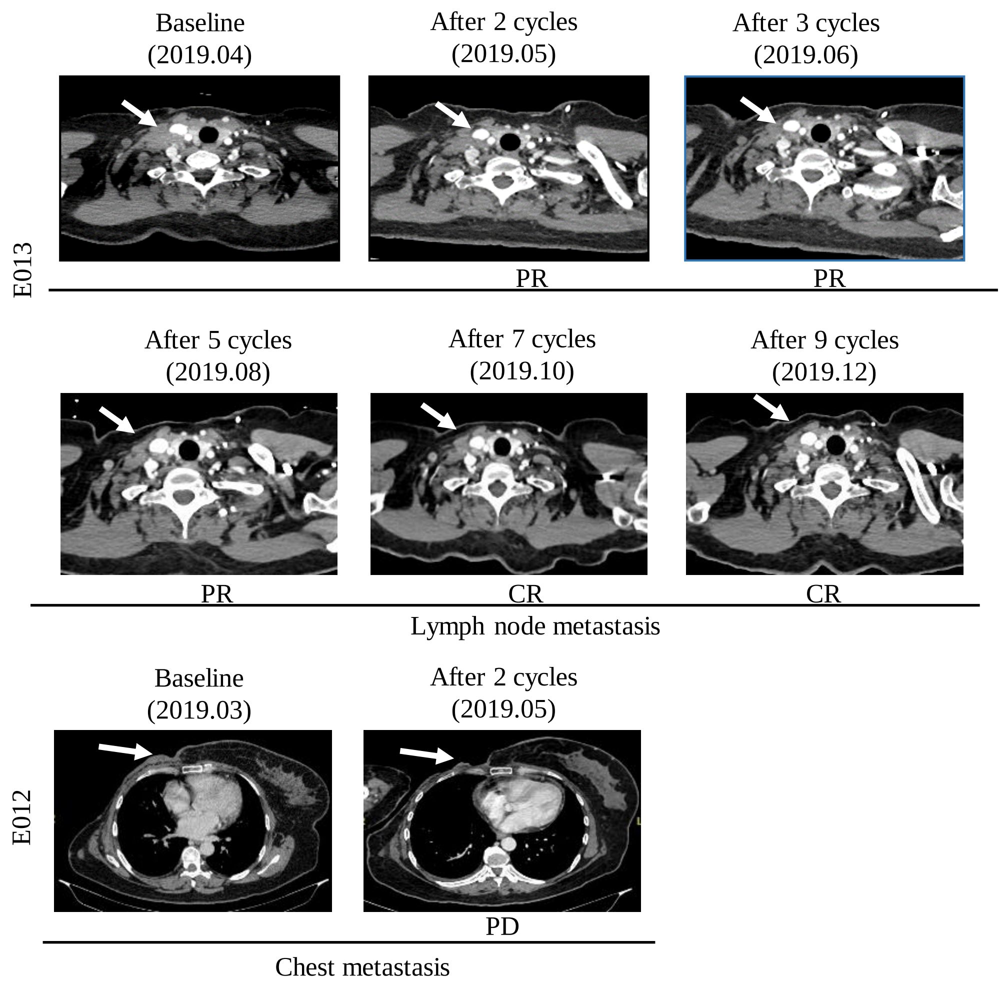

Baseline (2019.04)
After 2 cycles (2019.05)
After 3 cycles (2019.06)
E013
PR
PR
After 5 cycles (2019.08)
After 7 cycles (2019.10)
After 9 cycles (2019.12)
PR
CR
CR
Lymph node metastasis
Baseline (2019.03)
After 2 cycles (2019.05)
E012
PD
Chest metastasis

f

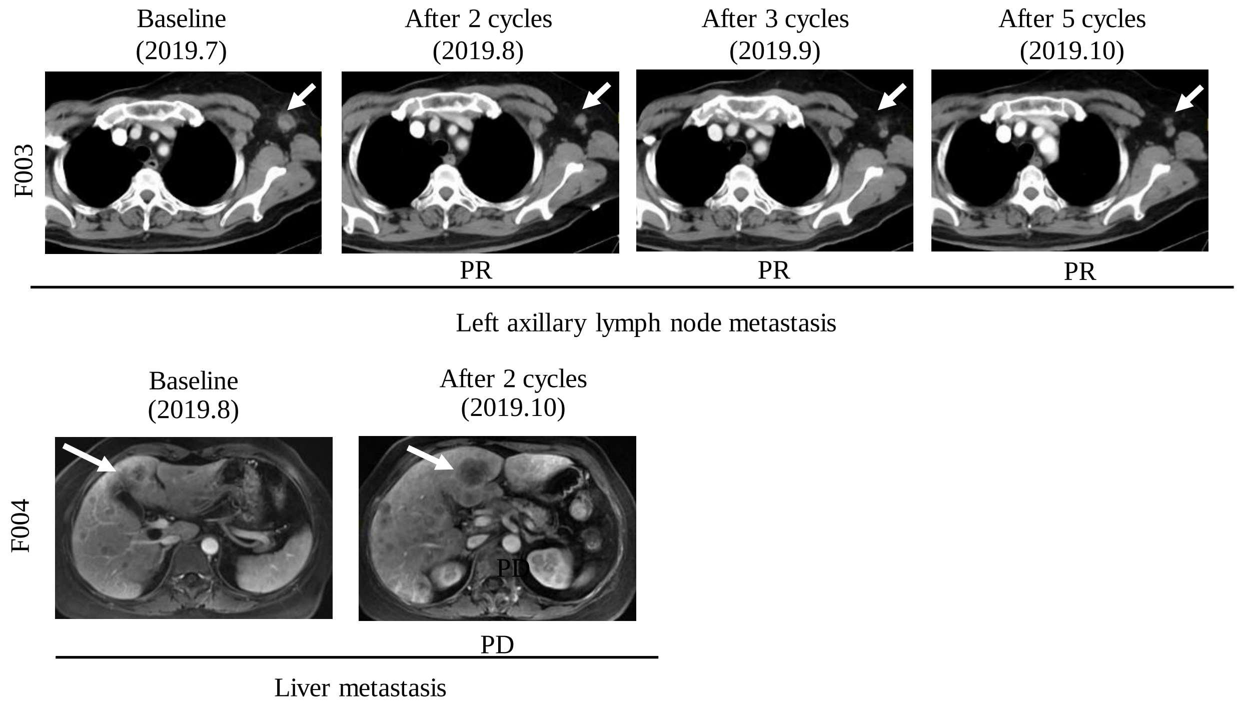

Baseline (2019.7)
After 2 cycles (2019.8)
After 3 cycles (2019.9)
After 5 cycles (2019.10)
F003
PR
PR
PR
Left axillary lymph node metastasis
Baseline (2019.8)
After 2 cycles (2019.10)
F004
PD
PD
Liver metastasis

g

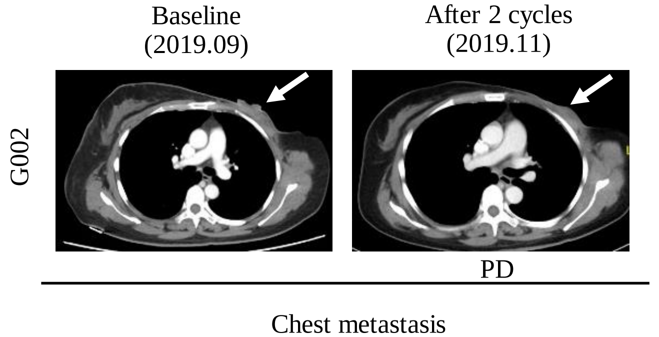

Baseline (2019.09)
After 2 cycles (2019.11)
G002
PD
Chest metastasis
