# Supplementary material for: Molecular subtyping and genomic profiling expand precision medicine in refractory metastatic triple-negative breast cancer: the FUTURE trial
Source: Cell Res. 2020 Jul 27;31(2):178–86. doi: 10.1038/s41422-020-0375-9 (PMC8027015; doi:10.1038/s41422-020-0375-9)
Supplement: Supplementary file 5 — Supplementary information, Fig. S4 [file 41422_2020_375_MOESM5_ESM.pdf]

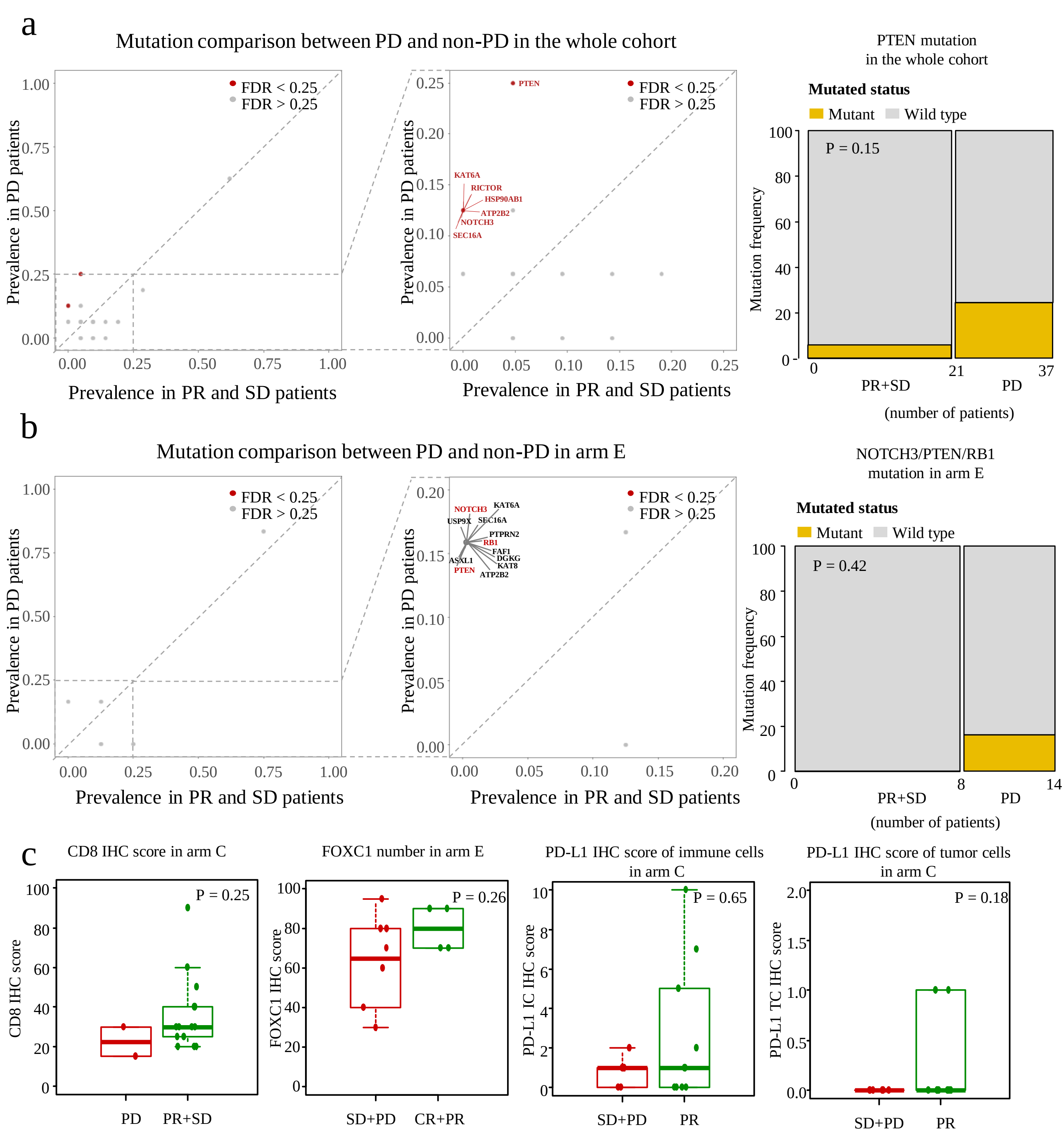


**Figure S4. Potential predictors of response in the whole cohort and arm C and E**

**(a and b)** Comparison of mutation frequency between PD, and non-PD patients (a) in the whole cohort, and (b) in arm E. **(c)** Comparison of CD8, FOXC1 and PD-L1 IHC score among different treatment efficacy groups.

Abbreviations: CR, complete response; PR, partial response; SD, stable disease; PD, progressive disease; FDR, false discovery rate; IHC: immunohistochemistry; TC: tumor cell; IC: immune cell.
