# Supplementary material for: Molecular subtyping and genomic profiling expand precision medicine in refractory metastatic triple-negative breast cancer: the FUTURE trial
Source: Cell Res. 2020 Jul 27;31(2):178–86. doi: 10.1038/s41422-020-0375-9 (PMC8027015; doi:10.1038/s41422-020-0375-9)
Supplement: Supplementary file 7 — Supplementary information, Fig. S6 [file 41422_2020_375_MOESM7_ESM.pdf]

a

B001

Characteristics	
**Age**	53
**Stage at diagnosis**	non-IV
**ECOG**	1
**Metastatic organ**	Lung, Liver, bone
**No. of previous lines of therapy**	5
**Drugs that have been used**	Anthracyclines, paclitaxel, carboplatin, vinorelbine, capecitabine, gemcitabine, tegafur and apatinib
**IHC results**	AR (+, 90%)、**CD8 (+, 25%)**、FOXC1 (-)

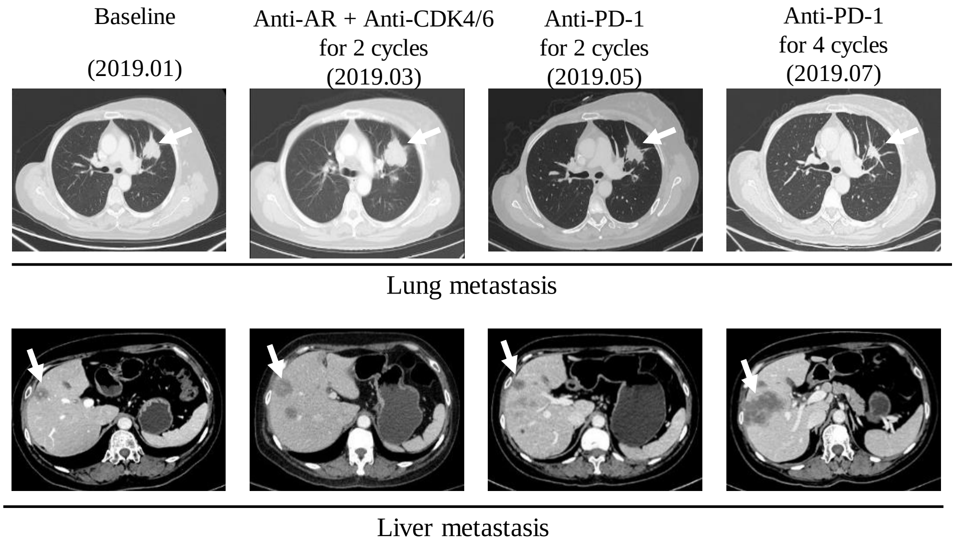

-	PD	SD	PD

b

B002

Characteristics	
**Age**	48
**Stage at diagnosis**	non-IV
**ECOG**	1
**Metastatic organ**	Lung
**No. of previous lines of therapy**	5
**Drugs that have been used**	Anthracyclines, paclitaxel, carboplatin, vinorelbine, gemcitabine, capecitabine
**IHC results**	AR3(+, 90%)、CD8 (-)、FOXC1 (-)
**SCNV**	**FGFR1 amplification**

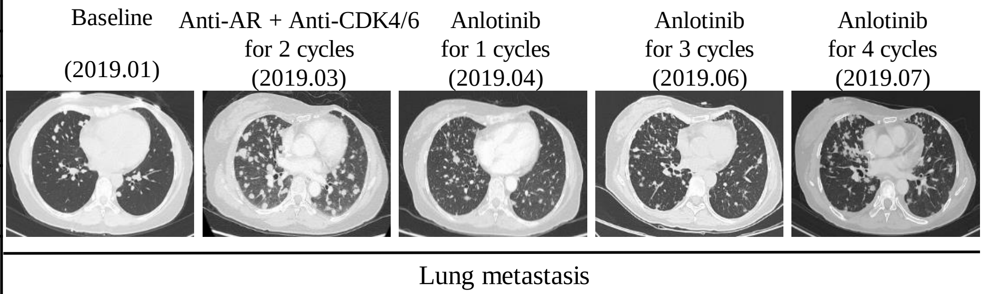

-	PD	PR	SD	PD

Figure S6. Patients benefited from other targets except for anti-AR plus anti-CDK 4/6 in arm B

(a) One patient with AR (+) and CD8 (+, 25%) benefited from anti-PD-1 therapy after progressed from therapy of arm B; **(b)** One patient with FGFR1 amplification benefited from anlotinib therapy after progressed from therapy of arm B

Abbreviations: PR, partial response; SD, stable disease; PD, progressive disease; ECOG, Eastern Cooperative Oncology Group.
