# Supplementary material for: Molecular subtyping and genomic profiling expand precision medicine in refractory metastatic triple-negative breast cancer: the FUTURE trial
Source: Cell Res. 2020 Jul 27;31(2):178–86. doi: 10.1038/s41422-020-0375-9 (PMC8027015; doi:10.1038/s41422-020-0375-9)
Supplement: Supplementary file 8 — Supplementary information, Fig. S7 [file 41422_2020_375_MOESM8_ESM.pdf]

a

D001

Baseline (2018.11) | After 2 cycles (2019.01)

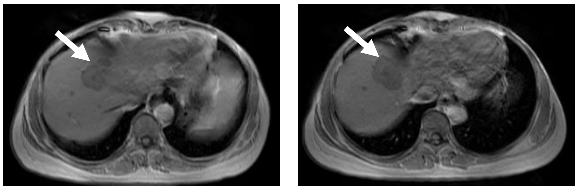

PD

Liver metastasis

Characteristics	
**Age**	48
**Stage at diagnosis**	Non-IV
**ECOG**	1
**Metastatic organ**	Liver
**No. of previous lines of therapy**	2
**Drugs that have been used**	Anthracyclines, paclitaxel, carboplatin, vinorelbine and capecitabine
**IHC results**	AR (+, 80%)、CD8 (+, 10%)、FOXC1(-)

**FUSCC NGS panel**			
Variants type	Gene	Site	VAF (%)
Pathological Germline mutation	*BRCA2*	NM_000059:exon11:c.5718_5719del:p.N1906fs	-
Somatic mutation	*AFDN*	NM_001291964:exon13:c.G1553C:p.R518T	17.4
	FAM47C	NM_001013736:exon1:c.G1339T:p.V447L	10.9
	MAST2	NM_015112:exon29:c.G4306A:p.E1436K	10.5
	PLK2	NM_006622:exon11:c.G1468C:p.D490H	11.3
	EHMT2	NM_001289413:exon17:c.G2474A:p.R825H	2.5
	FASN	NM_004104:exon42:c.G7153C:p.E2385Q	10.9
	NR1H2	NM_007121:exon3:c.C35A:p.P12H	2.9
	ARID1B	NM_017519:exon1:c.511_521del:p.A171fs	14.7

b

D002

Baseline (2018.11) | After 2 cycles (2019.01)

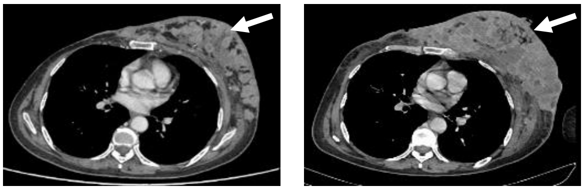

PD

Chest metastasis

Characteristics	
**Age**	56
**Stage at diagnosis**	IV
**ECOG**	1
**Metastatic organ**	Breast, lung, bone, lymph nodes, right chest
**No. of previous lines of therapy**	3
**Drugs that have been used**	Anthracyclines, paclitaxel, carboplatin, vinorelbine and capecitabine
**IHC results**	AR (-)、CD8 （+, 10%)、FOXC1 (+, 50%)

**FUSCC NGS panel**			
Variants type	Gene	Site	VAF (%)
Pathological Germline mutation	*BRCA1*	NM_007294:exon21:c.G5345A:p.W1782X	-
Somatic mutation	*RICTOR*	NM_152756:exon23:c.G2323A:p.D775N	15.4
	CYP2C8	NM_000770:exon1:c.G4T:p.E2X	3.2
	MAP2K4	NM_003010:exon1:c.G3T:p.M1I	3.8
	MAP2K4	NM_003010:exon2:c.C212A:p.P71Q	16.7
	BMX	NM_001721:exon15:c.C1534A:p.Q512K	7.3

Figure S7. Genomic features of TNBCs in arm D

(a-b) Clinical characteristics and genomic landscape of D001(a) and D002 (b).

Abbreviations: PD, progressive disease; IHC, immunohistochemistry
